# Comparative Functional Analysis of the Caenorhabditis elegans and Drosophila melanogaster Proteomes

**DOI:** 10.1371/journal.pbio.1000048

**Published:** 2009-03-03

**Authors:** Sabine P Schrimpf, Manuel Weiss, Lukas Reiter, Christian H Ahrens, Marko Jovanovic, Johan Malmström, Erich Brunner, Sonali Mohanty, Martin J Lercher, Peter E Hunziker, Ruedi Aebersold, Christian von Mering, Michael O Hengartner

**Affiliations:** 1 Institute of Molecular Biology, University of Zurich, Zurich, Switzerland; 2 Center for Model Organism Proteomes, University of Zurich, Zurich, Switzerland; 3 PhD Program in Molecular Life Sciences, University of Zurich, Zurich, Switzerland; 4 Institute of Molecular Systems Biology, Swiss Federal Institute of Technology Zurich, Zurich, Switzerland; 5 Functional Genomics Center, University of Zurich and Swiss Federal Institute of Technology Zurich, Zurich, Switzerland; 6 Institute of Informatics, University of Düsseldorf, Düsseldorf, Germany; 7 Institute for Systems Biology, Seattle, Washington, United States of America; 8 Swiss Institute of Bioinformatics, University of Zurich, Zurich, Switzerland; University of California San Francisco and Howard Hughes Medical Institute, United States of America

## Abstract

The nematode Caenorhabditis elegans is a popular model system in genetics, not least because a majority of human disease genes are conserved in C. elegans. To generate a comprehensive inventory of its expressed proteome, we performed extensive shotgun proteomics and identified more than half of all predicted C. elegans proteins. This allowed us to confirm and extend genome annotations, characterize the role of operons in C. elegans, and semiquantitatively infer abundance levels for thousands of proteins. Furthermore, for the first time to our knowledge, we were able to compare two animal proteomes (C. elegans and Drosophila melanogaster). We found that the abundances of orthologous proteins in metazoans correlate remarkably well, better than protein abundance versus transcript abundance within each organism or transcript abundances across organisms; this suggests that changes in transcript abundance may have been partially offset during evolution by opposing changes in protein abundance.

## Author Summary

Proteins are the active players that execute the genetic program of a cell, and their levels and interactions are precisely controlled. Routinely monitoring thousands of proteins is difficult, as they can be present at vastly different abundances, come with various sizes, shapes, and charge, and have a more complex alphabet of twenty “letters,” in contrast to the four letters of the genome itself. Here, we used mass spectrometry to extensively characterize the proteins of a popular model organism, the nematode Caenorhabditis elegans. Together with previous data from the fruit fly Drosophila melanogaster, this allows us to compare the protein levels of two animals on a global scale. Surprisingly, we find that individual protein abundance is highly conserved between the two species. So, although worms and flies look very different, they need similar amounts of each conserved, orthologous protein. Because many C. elegans and D. melanogaster proteins also have counterparts in humans, our results suggest that similar rules may apply to our own proteins.

## Abstract 3

A quantitative comparison of two animal proteomes shows a striking correlation of protein abundance levels, a better correlation than transcript levels. Are the latter more variable during evolution?

## Introduction

The rapid lifecycle, small size, reproducible development, and ease of cultivation in the laboratory have made Caenorhabditis elegans an important experimental system for biological studies. Numerous human disease-related genes (e.g., related to cancer or neurological diseases) have orthologs in the worm [1]. Sequencing and annotation of its genome has revealed more than 19,000 genes [2] coding for more than 22,000 proteins, including splice variants. Extensive systematic studies of gene function have been performed. However, to completely understand complex biological processes such as development, aging, or disease, the analysis of the proteome—i.e., the entire set of the expressed proteins—is becoming increasingly important. Knowledge of the complete sequence of a genome is a necessary prerequisite for proteomics, but the DNA sequence itself does not reveal which proteins are actually expressed when, where, and to what level. Furthermore, in contrast to the genome, the proteome is changing under different biological conditions. Although for many years, transcriptome data (i.e., the collection of transcribed mRNAs) has been used to approximate the proteome, a number of studies have demonstrated that the correlation between mRNA and protein abundance is surprisingly low [3–5] because of posttranscriptional regulation and variable protein half-lives. The analysis of the proteome is therefore a key method to provide systems-level information about protein function in time and space, and to obtain a concise view of biological processes. In the case of C. elegans, previous analyses of the proteome were either limited in scope and coverage [6,7], or largely focused on improving genome annotation [8], with the biggest C. elegans proteome dataset published so far encompassing 6,779 proteins [8].

To generate a comprehensive, deeply sampled C. elegans proteome database that can be used for quantitative proteome analysis, we applied subcellular and biochemical fractionation methods to the worm proteins, performed tryptic digests, separated the resulting peptides using a variety of techniques, and identified the peptides by mass spectrometry (MS). This resulted in a unique global view on the expression status of the C. elegans proteome. We identified a number of protein features and functions that are underrepresented in the expressed proteome, likely representing specialized functional systems expressed only in a small subset of cells and/or developmental stages. We demonstrate the importance of proteomics data towards improved genome annotation. Finally, we compared the proteome data with similar data from the fruit fly Drosophila melanogaster. The latter comparison provided—for the first time to our knowledge—an overview of the expressed “core animal proteome,” which should arguably become the initial focus for monitoring the basic metazoan cellular machinery in the future.

## Results

### Protein Identifications

**Figure 1:**
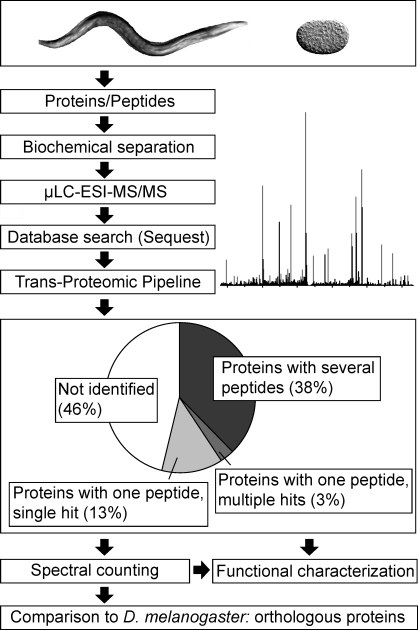
Workflow of the C. elegans Proteome Analysis Proteins and peptides were isolated from whole worm or egg homogenates, and separated biochemically. Peptides were identified by μLC-ESI-MS/MS and database searches, and validated using the Trans-Proteomic Pipeline [62]. We detected peptides for 10,631 different gene loci, which corresponds to 54% of the predicted gene loci in WormBase WS140 (19,735 gene loci). For 7,476 gene loci, more than one peptide was identified; for 580 gene loci, a single peptide was identified independently multiple times; for 2,575 gene loci, a single peptide was identified; and 9,104 gene loci were not covered at all.

To identify C. elegans proteins, we collected worms at various developmental stages and homogenized whole animals and eggs to isolate the proteins. Their tryptic peptides were separated using strong cation exchange chromatography (SCX), in several cases after labeling them with isotope-coded affinity tags (ICAT) [9] to reduce sample complexity, or by isoelectric focusing (applying free-flow electrophoresis and immobilized pH gradient strips). The peptides were finally identified using microcapillary liquid chromatography–electrospray ionization–tandem MS (μLC-ESI-MS/MS). With this extensive shotgun proteomics approach, we identified 10,977 different proteins, including splice variants, via 84,962 nonredundant peptide identifications (Table S1; 759,320 peptide identifications were obtained in total). We identified 10,631 gene loci, corresponding to 54% of the gene loci in WormBase (WS140: 19,735 loci). Of these, 7,476 loci (38%) were detected via several distinct peptides, 580 (3%) were detected via the same peptide more than once, and 2,575 (13%) were detected only via a single peptide identification (Figure 1). When considering individual annotated exons (irrespective of their various splicing contexts), our peptide data covered 28.2% of the 129,047 exons contained in WormBase.

Protein identification from MS peptide spectra is prone to false-positive assignments, and we employed strict search cutoffs using PeptideProphet (see Materials and Methods). To independently estimate our false discovery rate (FDR), in particular for identifications based on a single peptide spectrum (“single hits”), we first took advantage of one of our experiments that used isoelectric focusing to fractionate peptides. In each peptide fraction, true-positive identifications should scatter around a narrow range of isoelectric points (pIs), whereas false-positive identifications should follow the background distribution in the database. This analysis, using computational predictions of pIs to check all peptides, yielded an estimated FDR of 35% for single hits in this particular experiment. Independently, a newly developed model based on a robust decoy search strategy yielded an upper limit for the FDR of single-hit identifications at around 63% for all combined experiments (L. Reiter, M. Claassen, S. P. Schrimpf, J. M. Buhmann, M. O. Hengartner, et al., unpublished data). By the latter method, multi-hit identifications were found to be much more reliable, resulting in an FDR of 7% in our study. Since almost half of all single-hit identifications do represent bona fide protein identifications, we chose to include single-hit identifications in our subsequent analyses. A separate analysis focusing on just these proteins alone showed that they often belonged to groups that were underrepresented in the complete dataset and are therefore presumably of low abundance in C. elegans (short, uncharacterized proteins and in particular those with seven transmembrane domains; also see below). This means that they do represent valuable information about which proteins are expressed at low levels in C. elegans. It should also be stressed that all conclusions reported below remained valid when single-hit identifications were excluded.

To assess whether proteins from sources other than C. elegans were present in our preparations, we focused on the bacteria on which the worms were feeding (Escherichia coli). We tested a single, representative experiment, encompassing 67 MS/MS analyses by searching the spectra against a combined C. elegans and E. coli database. A total of 1.3% of the protein identifications mapped to E. coli, among them 14 hits mapping to both organisms. However, for each of these 14 proteins, there was at least one additional C. elegans peptide identified, confirming that these overlapping detections did not influence the C. elegans results.

### Proteins Seen and Not Seen: Features and Functions

In order to characterize C. elegans proteins that were *not* detected, and that are therefore most likely expressed at particularly low levels, or in specialized cells or developmental stages only, we classified the entire predicted C. elegans proteome with respect to several aspects (length, pI, hydrophobicity, transmembrane topology, and functional annotation). This should reveal the nature of underrepresented proteins (with potentially more peripheral, or even worm-specific functions), and separate them from abundant proteins involved in basic cellular processes such as growth, metabolism, and information processing. It should also reveal potential technical limitations (proteins/peptides difficult to detect using our procedure), which is important to assess for future systematic uses of MS.

**Figure 2:**
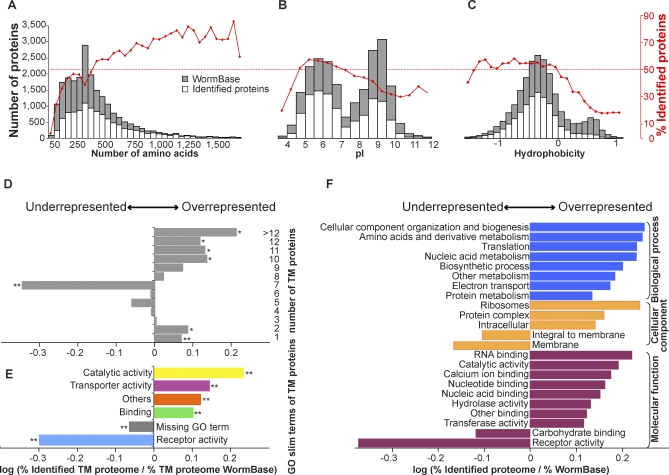
Classification of Detected Proteins (A–C) A bias analysis of the 10,977 identified proteins (including splice variants) in comparison to the 22,269 predicted proteins in WormBase (WS140) was performed for the parameters (A) length, (B) isoelectric point (pI), and (C) hydrophobicity. Red lines indicate the percentages of identified proteins in comparison to all C. elegans proteins in each bin. A value below 49% indicates fewer detections than expected; a value above 49% indicates more detections than expected. (D and E) Over- and underrepresentations of transmembrane (TM) proteins (D) and their functional classes (E) in our experimental dataset. Statistically significant categories are labeled with asterisks: *p-*values better than 0.05 are indicated by a single asterisk (*); *p*-values better than 1E−4 are indicated by double asterisks (**). The proportion of proteins with transmembrane helices was 36.5% in WormBase, and 30.5% in our proteome dataset. (F) The global functional GO slim analysis for all proteins showed statistically significant over- or underrepresentations in the categories “biological process,” “cellular component,” and “molecular function.” We used abbreviated terms for three categories (GO:0006139, GO:0008152, and GO:0005488).

Our bias analyses revealed an underrepresentation of proteins shorter than 400 amino acids (Figure 2A) and of proteins with basic pIs (Figure 2B). A similar bias has previously been observed for D. melanogaster [10]. The underrepresentation of basic proteins was partly to be expected, due to our isoelectric focusing experiments, which centered on the pH range 3–7. The underrepresentation of short (low molecular weight) proteins might be caused by a generally higher prevalence of spurious gene predictions among short genes, and also by a lower probability of detecting one of the few tryptic peptides generated by short proteins. We observed a bimodal distribution of hydrophobicity values within the annotated set of all C. elegans proteins, and a strong underrepresentation of proteins in the second, high hydrophobicity peak in our dataset (Figure 2C). This second peak consists mostly of multipass transmembrane proteins (∼64% of these proteins have seven or more predicted transmembrane domains). To better understand how membrane association relates to protein abundance and detectability, we globally characterized WormBase proteins with respect to their content of transmembrane segments, using Phobius [11]. Overall, we found a notable underrepresentation of transmembrane proteins in our proteomics data, and decided to subdivide these proteins further according to the number of transmembrane sections and annotated functions as shown for other species [12,13] (Figure 2D and 2E). Remarkably, we found that the strongest underrepresentation is observed for proteins with seven transmembrane regions, in particular those annotated with the function “receptor activity.” This may point to a biological (rather than technical) explanation for the relative paucity of transmembrane proteins in our data: Seven-transmembrane chemosensory receptors are widespread in the C. elegans genome, but many of these are known to be expressed only in a small number of neurons each [14–16]. Because we assessed whole animals, those proteins might be too rare to be successfully detected. This general underrepresentation in our proteome data suggests similar sensory functions for other transmembrane proteins of hitherto unknown function that we also found to be of too low abundance to be detected.

Finally, we globally analyzed the functional classifications of all the detected proteins. We observed a clear bias towards proteins with known functions. The same bias was also observed for the D. melanogaster proteome [10]. A possible explanation could be that some of the undetected proteins with unknown functions are actually erroneous gene predictions or pseudogenes. It could also be a testament to the biases of previous studies: abundant proteins are easier to work with biochemically, and may therefore have obtained a functional annotation more easily. In total, our proteomics approach identified proteins belonging to 125 out of the 127 Gene Ontology (GO) slim categories defined for WormBase. The global GO slim analyses confirmed the underrepresentation of proteins with receptor activity mentioned above, and of “membrane” or “integral to membrane” proteins in general (Figure 2F).

### Improving Genome Annotation

**Figure 3:**
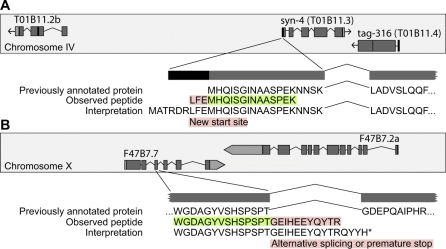
Improved Genome Annotation via Novel Peptide Identifications Examples of novel peptides obtained from genomic searches against a six-frame translation of the C. elegans genome, and the region where they match to the genome. (A) The novel peptide sequence LFEMHQISGINAASPEK suggests an alternative translational start site for the protein SYN-4 (T01B11.3). The sequence predicted to code for this peptide extends upstream of the annotated translational start site. An alternative start codon can be found further upstream in the same reading frame. (B) A peptide points at a novel splice variant that was identified for the gene F47B7.7. The peptide WGDAGYVSHSPSPTGEIHEEYQYTR extends an existing annotated exon into the downstream intron, resulting either in the selection of an alternative 5′ splice site downstream of the peptide, or in intron retention, which would result in an early translation stop (shown).

Large-scale proteome analyses (such as ours) represent an important cornerstone for an improved genome annotation. In WormBase (WS160), 4,987 gene loci were still listed with the gene status “predicted” only, i.e., without any supporting transcript data (expressed sequence tag [EST], mRNA). We experimentally confirmed the protein expression of 1,062 of these predicted genes (among them, more than 40% via multiple peptide detections). As was the case for the whole proteome, this subset was enriched for proteins with GO slim annotations (45% in our dataset, as compared to 38% expected for this subset in WormBase; *p*-value: 4.65E−08). Apart from these gene confirmations, our C. elegans proteomics dataset contains numerous spectra originating from nonannotated regions in the worm genome. In computationally intensive analyses, we are identifying these by searching our data against six-frame translations of the genome, and filtering the results for high confidence spectra that map to nonannotated regions. For example, from one particular experiment, we identified 78 likely novel peptides. Two of these are illustrated in Figure 3 (the corresponding MS/MS spectra are provided as Figure S1). These data suggest an alternative translational start site for the protein SYN-4 (T01B11.3; Figure 3A): the observed peptide is located upstream of the annotated translational start site, and only partially overlaps with the currently annotated protein sequence. The second example demonstrates a novel splice variant for the gene F47B7.7 (Figure 3B). In this case, we identified a peptide that extends an existing annotated exon downstream, in the correct frame. These and similar analyses, suggesting altered or new gene models, are computationally very intensive and were not yet completed at the time of submission. Furthermore, due to the increased search space when searching proteomics against the genome, extra scrutiny is needed when interpreting each reannotation instance, and additional experimental data should probably be taken into account before fully accepting these gene annotation changes.

### Operons

**Figure 4:**
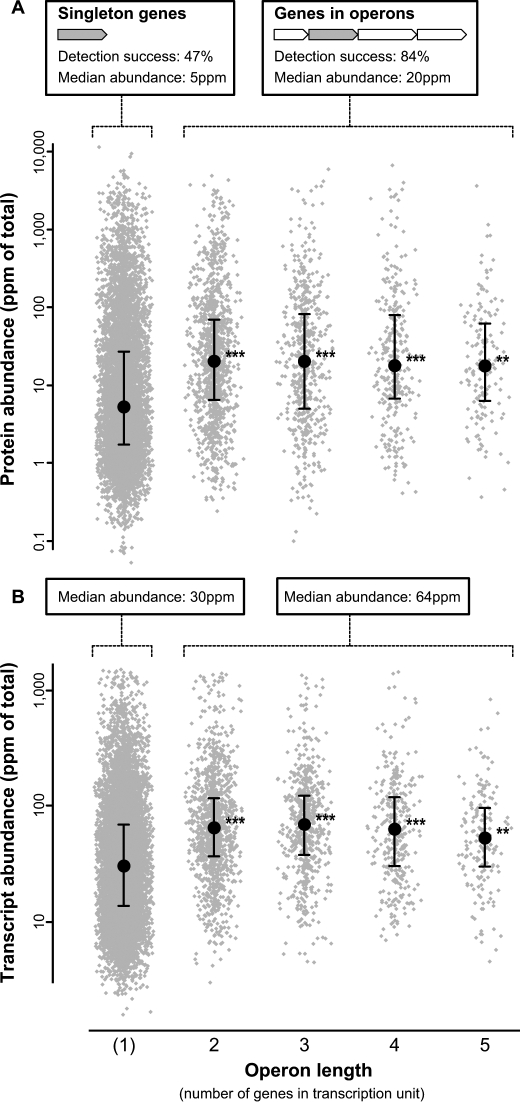
Operon Genes Are More Highly Expressed Than Singleton Genes (A) Proteins whose genes are organized in operons were identified more frequently (84%) and more abundantly (median expression: 20 ppm) compared to proteins encoded by individually transcribed genes (47%; 5 ppm). *p-*values: double asterisks (**) indicate better than 1E−10; triple asterisks (***) indicate better than 1E−15. (B) A similar result is obtained when analyzing Affymetrix data instead (albeit with a smaller abundance difference). In both panels, the left-most data column encompasses singleton genes (i.e., not in operons), and the four columns to the right encompass genes in operons of various lengths. Medians are indicated as black dots, and whiskers encompass the range from 25% to 75% of values.

C. elegans and its relatives are unique among characterized metazoans in that a large number of their genes are organized into operons (multicistronic transcription units, containing up to eight genes that are strictly coexpressed [17,18]). Following transcription, the primary transcript is split up through a unique trans-splicing mechanism, and the individual open reading frames are subsequently translated separately into distinct mature proteins. In order to assess the potential influence of operon structure on the regulation and abundance of proteins, we studied the expression status of genes in operons, compared to individually transcribed genes. Although an absolute quantification of protein levels is not possible with our shotgun approach, we performed a semiquantitative analysis based on spectral counting [19–23]. Surprisingly, we observed that proteins encoded by operons are expressed far more strongly than those encoded by individually transcribed genes: we observed 84% of the former, with a median relative abundance of 20 ppm (parts per million of total protein molecules), but only 47% of the latter with a median relative abundance of 5 ppm (Figure 4A). The same tendency was found when analyzing publicly available transcript-abundance data (Figure 4B). This striking observation confirms that operons are preferentially made up of genes that are strongly transcribed, and we now establish that this is reflected also at the protein level: operon proteins, on average, are more than 3-fold more abundant than proteins from single-gene transcripts. Apart from grouping strongly expressed proteins, operons are also expected to facilitate coordinated regulation of their constituent genes. We assessed whether this is the case by searching for operons that were either fully expressed (i.e., all encoded proteins detectable) or silenced (none or very few of the encoded proteins detectable). Indeed, we found significantly more operons of both types than expected by chance, as illustrated for operons of lengths 4 to 6 (Figure S2). In principle, our observations could be stemming from a limited selection of tissues only, for example from the hermaphrodite germline, where operons are thought to be strongly expressed during oogenesis [24]. However, we observed that operon proteins are more abundant even in dauer and L1-stage larvae, which both should have very little germline material (Figure S3). We further checked whether our observation could be explained by systematic differences in length or transmembrane segments of operon proteins. Although we did observe slight differences in length and transmembrane content—operon proteins are on average 11% longer, and transmembrane proteins are 40% less frequent—these differences were not sufficient to explain the increased abundance of operon proteins (unpublished data). Together, our observations indicate, for the first time, that operons in C. elegans ensure the coordinated regulation of highly expressed proteins.

### Comparison to the D. melanogaster Proteome Dataset

**Figure 5:**
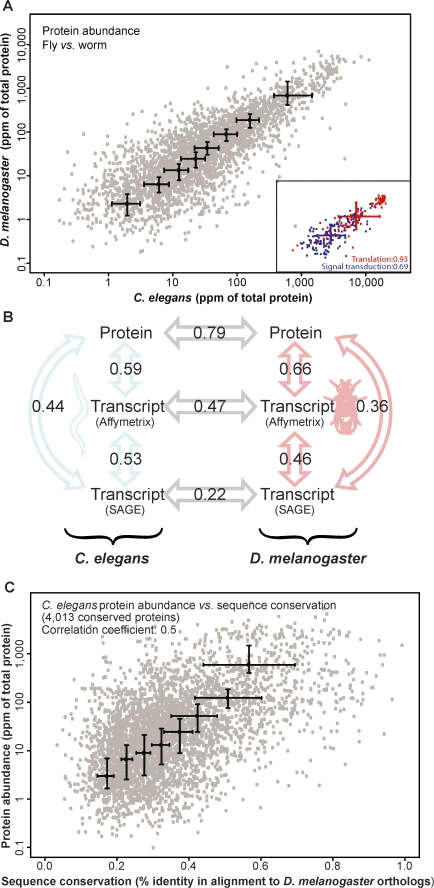
Interspecies Comparative Proteomics of Orthologous Proteins in C. elegans and D. melanogaster (A) Protein abundances deduced from spectral counting of 2,695 pairs of orthologs from both species are shown. Medians of equal-sized bins are indicated as crosses; whiskers encompass the range from 25% to 75% of values. The distribution of the orthologs (dots) is indicated in the background. The distribution and correlation coefficients of proteins involved in signal transduction and translation are shown in the inset. (B) The correlation coefficient of *R_S_* = 0.79 between the two species is higher than that of the comparison between protein and transcript abundance within the organisms, based on SAGE or Affymetrix data. (C) For C. elegans, we plotted protein abundance versus sequence conservation (the latter determined by alignment with the D. melanogaster orthologs). All correlation coefficients are rank-based with *p-*values better than 2.2E−16.

In this study, we had the unique opportunity to compare large-scale proteome datasets from two different animal species, owing to the recent publication of the D. melanogaster proteome [10] (http://www.mop.uzh.ch/peptideatlas/; previous work in D. melanogaster had mainly focused on protein–protein interactions or subproteomes only [25,26]). We performed spectral counting for both organisms to obtain semiquantitative measurements of protein abundance, and compared these to published mRNA expression data derived from Affymetrix [27,28] and serial analysis of gene expression (SAGE) platforms [27,29]. In the C. elegans and D. melanogaster proteomes, 2,695 pairs of orthologs were identified for which all three types of data were available. Surprisingly, we observed that orthologs showed a strong correlation in protein abundances across the two organisms, despite more than 600 million years of separate evolution (Spearman rank correlation *R_S_* = 0.79; Figure 5A). Notably, this biological correlation at the protein level between the two species is even higher than the within-species correlation between protein and transcript abundances (within C. elegans: *R_S_* = 0.59 and 0.44 for protein-Affymetrix and protein-SAGE, respectively; within D. melanogaster: *R_S_* = 0.66 and 0.36, respectively). In contrast to the protein-level correlation, the abundance correlations at the transcript level between the two species were also rather low (Figure 5B). Interestingly, the overall protein-abundance correlations are not equally tight across functional categories: the highest correlation was observed for the functional category “translation” (*R_S_* = 0.93) and the lowest for the category “regulation of biological process” (*R_S_* = 0.65).

Despite the fact that it is difficult to compare tissues and developmental stages across organisms, our analysis provides a first insight into the evolutionary behavior of animal proteins over long time scales. It is important to point out that for all six data points, several developmental stages and/or tissues had been mixed, but that these were not, of course, always directly equivalent and comparable between the two organisms. However, many of the ancient animal orthologs that we studied here can be expected to be expressed similarly across many cell types and stages, and we thus attempted to capture an organism-wide “average” proteome for both animals. That notwithstanding, we also repeated the analysis for one set of samples that is arguably more directly comparable: mixed staged embryos sampled in both D. melanogaster and C. elegans at the proteome and at the transcript levels (Figure S4). Here again, we saw that protein abundances correlated far better (*R_S_* = 0.70) across organisms than transcript abundances (*R_S_* = 0.50).

Another potential complication for our analysis lies in the technique of spectral counting. Individual tryptic peptides are known to ionize and be detected with widely differing efficiencies in mass spectrometers. Although protein conservation between C. elegans and D. melanogaster is low (∼40% sequence identity), a higher-than-expected abundance correlation might still result if equivalent peptides in both organisms were correlated in their suitability for MS. We assessed the extent of this effect by making the spectral counts independent: For any given section in the alignment of two orthologs, only one of the proteins was allowed to generate peptide counts; these sections were alternated across the length of the alignment, effectively reducing the data by half. As expected, this lowered the abundance correlation, but not by much (*R_S_* = 0.68). Importantly, the resulting correlation is still much higher than the correlation of transcript abundances across organisms (Figure S5).

Since one of our original interests was to characterize the “core animal proteome,” we also analyzed lower-coverage datasets from two additional organisms: Saccharomyces cerevisiae [30] and Mus musculus ([21]) (for the latter, we additionally included plasma data from PeptideAtlas; http://www.peptideatlas.org/). Comparative proteomics using multiple organisms has recently become popular, for example in bacteria [31], but has not yet been possible for animals. We searched for groups of orthologs that were detected in all four organisms; these would constitute the universally detectable eukaryotic proteome core. We found 847 such proteins, mostly from information-processing and metabolism genes. Conversely, we found 1,287 proteins to be detectable in all three animals, but not in yeast. This latter set might be considered the specific core of multicellular animal proteomes. However, it is clear that neither of these sets is complete, as of yet, mostly due to low coverage in mouse.

### Expression Levels of Duplicated Genes

Our protein-abundance estimates from two organisms also allowed us to study in more detail the fate of duplicated genes. Here, of particular interest, are cases in which a gene family has duplicated in one lineage (fly or worm), but not in the other. It is known that long-term retention of duplicated gene copies requires neo- or subfunctionalization [32–34], but it is unclear what consequences this has for overall protein-abundance levels. We found that when averaging over all cases of lineage-specific gene duplications (Figure S6), the abundance of duplicated genes is significantly lower than that of their nonduplicated counterparts in the other lineage. Strikingly, however, when all the duplicated genes of a given gene family are pooled, they tend to add up again to the original abundance of the nonduplicated counterpart (Figure S6).

## Discussion

We describe here a comprehensive inventory of C. elegans proteins, the functional characterization of this inventory, and the first-ever comparison of two such inventories between two model animals (“comparative proteomics”). Although some subsets of the proteome are more difficult to analyze (e.g., the membrane compartment), we achieved a relatively thorough representation of the genome, where the major exceptions can be explained biologically. For example, the systematic underrepresentation of seven-transmembrane proteins appears to be caused mainly by G protein–coupled receptors. The putative chemoreceptor gene families in C. elegans encompass about 7% of its total genome [35], and many are thought to be expressed only in a few neurons each [14–16]. Despite their generally low abundance, we did identify 172 seven-transmembrane receptor proteins, showing that they are, in principle, amenable to high-throughput MS analysis (this is relevant, for example, for screens of putative therapeutic targets).

We also demonstrated that a whole-proteome analysis of a model organism can contribute to an improved genome annotation. First, we experimentally confirmed the expression of 1,062 predicted genes for which no transcript data were available, but for which our proteome data allowed the extraction of a first rough expression pattern. Second, we identified novel peptides from spectra that could not be matched to annotated gene models, suggesting a way to more precisely map open reading frames and splice isoforms to the genome.

With respect to genome organization, we found that, in C. elegans, genes in operons are far more consistently and more strongly expressed than individually transcribed genes. In principle, this observation could be an artifact of genome annotation—if a disproportionally large number of annotated nonoperon genes were misannotations that are biologically meaningless. This is highly unlikely, however, since more than 6,000 such misannotations would be needed to reconcile the observed differences. Instead, it is likely that operons in C. elegans indeed serve to group strongly expressed genes into coregulated transcription units. Another question that arises is whether these genes were highly expressed even *before* they were grouped into operons, which would hint at a possible selective advantage for the grouping (e.g., to enable more efficient, more reliable, or more uniform transcription of genes whose products are in high demand). This is difficult to address conclusively, but our comparison to D. melanogaster provides some information: we observe that orthologs of operon genes are more strongly expressed even in the fly (Figure S7), where they are not arranged in operons nor are even neighbors on the genome. If one assumes that the operons in C. elegans are the derived state, then the corresponding genes were indeed already strongly expressed before they formed operons.

The comparison of our data to the D. melanogaster proteome also sheds some light on an important evolutionary puzzle, namely the surprisingly low correlation between mRNA expression levels of orthologous genes across animal species [36,37], despite evidence for strong stabilizing selection against expression changes in experimental evolution [38]. We found that the abundances of orthologous proteins from worm and fly correlate well (*R_S_* = 0.79), far better than the corresponding abundances of mRNA transcripts (*R_S_* < 0.50; Figure 5B).

There are several possible explanations for this finding: First, sweeping changes within the transcriptional machineries in one or both organisms could have resulted in global differences in transcript abundance, whereas selection would have kept protein abundances at least partially stable. One candidate for such a mechanistic change could be, for example, the unique trans-splicing mechanism of nematodes. A second possible explanation might be that posttranslational regulation may have changed systematically, for example due to differences in developmental strategies, physiology, or life styles of the two animals. Here, possibly relevant changes include the fixed cell lineage of nematodes, differences in reproductive strategies, increased endurance in nematodes (dauer stage), or the constraints imposed on D. melanogaster because of its need for metamorphosis and its higher motility (flight).

However, in our view, the most parsimonious explanation might be that many changes in the transcriptome might be neutral, or at least nearly neutral [36]. Ultimately, it is the protein levels that are under selection. Protein levels are not only determined by mRNA abundance, but are equally affected by translation efficiencies, protein half-lives, and other factors. Genetic mutations resulting in small changes on any of these levels might persist for some time in a population, as long as their fitness effects are small (around 1/[2*N_e_*] or less). This might be sufficient time to allow for compensatory mutations either in the same gene or elsewhere in the genome, which would reconstitute optimal protein abundance through action on the same or another factor that influences protein abundance. Thus, changes in mRNA expression could be offset by opposite changes in translation rate or protein half-life, and vice versa. Over evolutionary time scales, such small changes may accumulate, resulting in appreciable changes of mRNA abundance, whereas protein abundance would remain roughly constant. This model is a generalization of the concept of compensatory mutations that explains the rapid divergence of some *cis*-regulatory nucleotide sequences despite the maintenance of stable transcript levels [39], or the conserved expression of assembled protein complexes despite variable expression patterns of their individual components [40].

The presence of several interacting levels of protein-abundance regulation also would explain another two of our observations: a wide variance of the number of mature proteins per transcript, and a correspondingly low correlation between protein and transcript abundance within an organism (interestingly, the latter correlation is quite similar between our C. elegans data and data published in yeast [41] [*R_S_* = 0.57]). Our data, in principle, provides an opportunity to study transcript features that would directly influence the ratio of proteins per transcript (and thereby potentially uncover novel mechanisms of translational regulation). However, when checking the influence of transcript length, GC content, or UTR length, we failed to detect correlations with protein/transcript ratios (unpublished data). We did observe a weak, but significant, positive correlation of our protein/transcript ratios and experimental protein half-life measurements of orthologous proteins in yeast [42] (unpublished data), suggesting that protein stability is indeed one of the factors determining the steady-state protein/transcript ratio.

We note that the most abundant proteins (often found in central pathways like energy metabolism or protein synthesis) also tend to be the ones that show the best abundance correlation between species. This may simply be the case because of a greater relative measurement accuracy for abundant proteins. However, highly expressed genes are also more likely to be housekeeping genes [43], and may thus be more likely to be under the same evolutionary pressures in different organisms. Strong and constant stabilizing selection is also consistent with our observation that amino acid sequences of more highly expressed proteins evolve more slowly (Figure 5C), mirroring the analogous observation for mRNA expression data [44].

When we stratify proteins by functional categories, we find that those involved in translation and in core metabolism are those with the most highly correlated abundances across species. These functional groups are also those where the coexpression between pairs of transcripts is most highly conserved across species [45]. Furthermore, the same categories also tend to show the best correlation *within* each organism, with respect to rank-correlation between transcripts and proteins (Table S2). We also find that the correlation between transcript and protein levels is particularly poor for genes that are presumably heavily regulated (the categories “signal transduction” or “transcriptional regulation”), arguing for abundant posttranscriptional regulation in these functional classes.

Proteins differ not only in their mean abundance, but also in the variance of this abundance among individuals (“noise”) [46]. Interestingly, whereas yeast proteins involved in translation also show low levels of noise [47], other groups of proteins found here to be conserved in their abundance between species (e.g., protein metabolism) are characterized by high protein expression noise [47]. Thus, it appears that abundance fluctuations on short time scales (within populations) are partially decoupled from fluctuations on long time scales (between species). However, as natural variation is the substrate of evolutionary change, we expect that changes in mRNA levels via compensatory mutations may occur faster in proteins that exhibit higher levels of noise; this remains to be tested in future studies.

Our comparative analysis underlines clearly the necessity and usefulness of quantitative proteome analyses, since these better reflect the abundance of the actual effectors of biological processes. Most likely, the actual conservation of protein levels is even higher than what we report here, due to the shortcomings of a simple spectral-counting procedure. In fact, comparisons across organisms might generally provide a good test scenario to improve spectral-counting algorithms or other proteomics algorithms: the higher the abundance correlation, the more precise the measurements (due to the high number of data points, and due to the quickly changing positions of tryptic cleavages, this is difficult to “over-train” by choosing biased parameters). With respect to the transcriptomics datasets that we used, the above test argued for a better quality of the Affymetrix data, as compared to SAGE, because the latter were seen to correlate less well across organisms. This is intriguing, and it may point to additional biases in the SAGE procedure (for example, due to the added molecular biology steps of cleavage and ligation) [48].

For those instances where orthologs were *not* found to be of similar abundance, one can speculate that this difference reflects differing roles (or even molecular functions) of the orthologs. Thus, these proteins are of particular interest when studying the evolutionary differences between species. Alternatively, differences in technical aspects for particular proteins might occur, such as shifted or absent trypsin cleavage sites or differences in protein solubility. Interestingly, we did not lose the observed interspecies correlation even for quite low-abundance proteins such as those involved in signal transduction (our measurements have a dynamic range of more than three orders of magnitude). This means that low-abundance measurements are still quantitative, at least to some degree.

In our analysis of gene families with lineage-specific duplications, we found that duplicated proteins generally have lower abundance than their nonduplicated counterparts, whereas the summed abundances per gene family remained roughly constant. This finding might be most parsimoniously explained by a prevalence of subfunctionalization among duplicated genes, although it is also consistent with other scenarios (e.g., complementarity of tissue expression domains, functional fine-tuning, or subfunctionalization followed by neofunctionalization [49]). Of course, protein abundances alone cannot directly inform us about any changes in the functions of duplicated genes. However, our finding does suggest that cases where an increased demand for protein product would provide the sole driving force behind gene copy retention are probably rare.

With our dataset, we established an inventory of where and how proteins of interest can be specifically accessed using MS. It enables the generation of a proteotypic peptide library (i.e., peptides in a protein sequence that are most likely to be consistently and confidently observed by current MS-based proteomics methods). This library in turn can be used for targeted analyses and comparative studies of expressed proteins [10,50–52] by spiking the samples to be analyzed with chemically synthesized proteotypic peptides, or by selected reaction monitoring (SRM) MS. Our C. elegans proteome dataset will be made publicly available within WormBase and will thus be useful for the entire C. elegans research community. In general, proteomics data like ours is closer to the biologically active players than transcriptomics data. It should therefore be increasingly used to investigate biological phenomena and mechanisms underlying disease pathogenesis such as neuronal degeneration and cancer development, and for the identification of conserved therapeutic target proteins.

## Materials and Methods

### C. elegans.

C. elegans wild-type strain N2 (Bristol) was grown on 9-cm nematode growth medium (NGM) agar plates seeded with a lawn of the E. coli strain OP50 or in 100 ml of liquid cultures in S-basal buffer in beveled flasks. Worms were harvested from plates or liquid culture, and separated from the bacteria by washing with water or sucrose flotation. For the collection of embryos, the worms were synchronized, and eggs were removed from agar plates or obtained from the hermaphrodites by bleaching. Worm and egg pellets were homogenized with glass beads (diameter of 212–300 μm; Sigma-Aldrich) in the ratio of 1:1:2 (worms:beads:buffer) in a cell disrupter (FastPrep FP120, Thermo Savant; Qbiogene) at 4 °C three times for 45 s at level 6. The buffer used was 50 mM Tris/HCl (pH 8.3), 5 mM EDTA, 8 M urea. After glass bead treatment, 0.125% SDS was added, and the homogenate was incubated for 1 h at room temperature (RT) to solubilize proteins. For other experiments, the worms or eggs were homogenized with glass beads in 50 mM Tris/HCl (pH 8.3), 5 mM EDTA, then 0.75% or 1% Rapigest (Waters) was added, the homogenate was heated at 95 °C for 5 min, and incubated at RT for 30–60 min with gentle agitation. Cell debris was removed by centrifugation, and the protein concentration was determined using the Bradford reagent (Sigma-Aldrich).

### Tandem mass spectrometry.

The peptides were subjected to reversed-phase capillary chromatography using a 75-μm × 8-cm self-packed C18 column (Magic C18; Michrom) at a flow rate of 250 nl/min. Peptides were eluted with a gradient between solvent A (5% ACN, 0.2% formic acid) and solvent B (80% ACN, 0.2% formic acid). The gradient was from 5% up to 45% solvent B within 69 min. The peptides were identified by CID (collision induced dissociation) on a Thermo-Finnigan ion trap mass spectrometer “LTQ”. Six dependent scans followed each survey scan. Raw data were converted into mzXML files and searched against a C. elegans database derived from the Wormpep database (http://www.wormbase.org, release WS140) using the Sequest program [53]. The search parameters used were two missed cleavage sites, two tryptic termini, a mass tolerance of 3 Da for the parent ion and 0.95 Da for the fragment ion, optional oxidized methionine, and depending on the experiment, modified cysteine. Peptide assignments were statistically validated at peptide level using PeptideProphet [54], and peptides with a probability score of 0.9 or higher and the proteins they belong to were selected. For the qualitative analysis of the proteome (Figure 2), peptides matching to more than one protein (such as duplicated tubulins or histones), or matching to several splice variants of a protein, were counted only once (for the first entry of the search results). For the quantitative analysis, however, such peptides were assigned fractionally (see below). From a total of 18 different experiments (Table S3), we identified 10,977 proteins from 10,631 gene loci (Table S1). The comparative analysis of the different protein parameters was also based on WS140. For technical reasons, all the information for the other functional analyses was extracted from release WS160 using WormMart (http://www.wormbase.org/biomart/martview). The FDR for single hits was estimated first based on an experiment in which isoelectric focusing of peptides was performed on an immobilized pH gradient strip (pH range 3–5.6), followed by subsequent analysis of computationally predicted pIs for each peptide identification, and second by a new model based on a decoy search strategy (L. Reiter, M. Claassen, S. P. Schrimpf, J. M. Buhmann, M. O. Hengartner, et al., unpublished data). To evaluate potential bacterial contamination in our dataset, one experiment was searched against a combined C. elegans (WormBase WS140) and E. coli (SPproteomes at the European Bioinformatics Institute [EBI], release 2005-03-19, 4,338 entries) database using the same search parameters as for the searches against the C. elegans database.

### Bias analysis of protein parameters.

After redundancy analysis, 22,269 distinct proteins (including splice variants, WormBase WS140) and 10,977 proteins in our dataset were compared for the bias analysis with respect to different protein parameters. Tools from the ExPASy Web site (http://www.expasy.ch) were used to calculate the pIs of proteins (protein parameter tool “protparams”) and their hydrophobicity (gravy computation “grand average hydrophobicity”). The statistical analysis shown in Figure S8 was carried out as described before [10]; the *p-*values for all parameter analyses were 1E−10 or better.

### Transmembrane domains and GO slim terms.

The number and orientation of transmembrane domains of the proteins in WormBase (WS160) and in our dataset were predicted using Phobius [11]. Only gene loci—not splice variants—were processed. Whenever transmembrane predictions differed for splice variants, the predictions for the longest splice variants were used. For the GO slim analysis, the GO terms listed in WormBase (WS160) were mapped onto higher-level terms using the GO slim guide (http://www.geneontology.org), with two exceptions: the terms “membrane” and “integral to membrane” were not mapped to the higher category term “cell,” but instead were retained. In Figures 2E and S9, we assigned the GO slim terms of the category “molecular function” to the predicted transmembrane proteins. For 412 proteins, there was more than one entry for molecular function. For the statistical analysis of the GO slim categories in Figure 2, we applied the Fisher exact test and included the Bonferroni correction for multiple testing. We plotted the log ratio of observed versus expected, using the proportions in WormBase as the expectation. The GO slim categories with a *p-*value better than 0.05 are shown (Figure 2E and 2F).

### Genome annotation.

We mined our dataset for nonannotated translated regions by preparing a whole-genome open reading frame database that was searched using the Sequest algorithm [53]. To do this, WormBase release WS160 was used to translate each chromosome into all six reading frames. Open reading frames longer than 20 amino acids were assembled into a database with headers containing the coordinates of the sequences on the genome. The resulting database contains 3,136,258 open reading frames and 132,018,220 amino acids. A subset of our data (experiment 15) obtained by isoelectric focusing, comprising approximately 304,000 MS/MS spectra, was searched at the Functional Genomics Center Zurich. We allowed fully tryptic peptides with up to two missed cleavages, and specified oxidized methionine as variable modification and carbamylated cysteine as static modification. The results were further analyzed with PeptideProphet [54], and 27,940 search hits with a PeptideProphet score greater than or equal to 0.95 were selected. From these, we removed 26,952 scans that also generated a hit against the normal Wormpep140 protein database with a score greater than 0.8. Of the remaining 988 spectra, 789 were further observed to exist in Wormpep178 or an E. coli database and were therefore omitted, resulting in a final set of 199 spectra belonging to 173 different peptides. For the resulting peptides, a theoretical pI value was calculated and compared to the mean pI of all peptides in the corresponding fraction. Only peptides with a delta pI value smaller than or equal to 0.5 were selected. This resulted in 78 distinct peptides.

### Operons.

WormMart (http://www.wormbase.org/biomart/martview) was used to extract operon architectures from WormBase release WS160. To test whether the coregulation of genes in operons would be detectable also at the level of translated proteins, operons were first divided into length classes (here, length is defined as the number of cotranscribed genes in each operon). For each length class, the fraction of operon genes was then determined for which at least one peptide was detected in at least one proteomics experiment. This fraction determines how many proteins should, on average, be detectable from a single operon if expression of the operon genes were truly independent (when assuming independence, the number of detections per operon should follow a binomial distribution, shown as grey lines in Figure S2). Applying the two-sided Kolmogorov-Smirnov test yielded *p-*values better than 1E−10. For the study of operons in specific stages (Figure S3), only the proteome data was analyzed, limited to the experiments done in these stages (with concomitantly reduced spectral counts).

### Semiquantitative interspecies proteomics comparison.

For the semiquantitative comparison between C. elegans and D. melanogaster proteomes, we used the STRING database and the Smith-Waterman similarity relations stored therein to compute orthologous groups [55]. This analysis retrieved 4,184 loci in C. elegans, and 4,302 in D. melanogaster. When working with orthology sets, each pair of orthologs was aligned with “muscle” [56], available from http://www.drive5.com/muscle/. The protein sequences used were extracted from WormBase WS160 and from FlyBase release 5.1 (http://flybase.org). Due to lineage-specific gene duplications, some proteins had several orthologs. For the interspecies abundance correlation comparison, we summed up the abundances in these cases.

We independently tested another source of orthology information, InParanoid [57], which resulted in slightly more orthologs but also in a somewhat lower interspecies abundance correlation (*R_S_* = 0.76 versus 0.79). Conversely, we also tested a stricter set of orthologs, to test for and exclude artifacts caused by potentially undetected paralogy. To conclusively separate orthologs from paralogs can be difficult, and this is the subject of intense study [58–60]. Therefore, we constructed a very strict set of orthologs by searching for reciprocal best matches between worm and fly, with the additional constraint that any extra homologs within these genomes had to exhibit no more than half the alignment score than the score *between* these organism (plus, the score between the organisms had to be 60 bits or higher). This strict set contained only 2,001 pairs of orthologs, and resulted in an interspecies abundance correlation of 0.80. This shows that our high correlation is not caused, or affected, by the presence of paralogs in the comparison.

We calculated the relative abundance of a protein by counting how often any of its amino acids had been identified in any peptide, divided by the total number of amino acids of the protein sequence. A length restriction to peptides with ≥7 and ≤40 amino acids (modified from [22]) was applied.


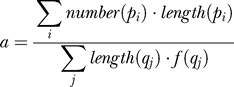


where *a* = protein abundance, *p* = identified peptides, *q* = tryptic peptides (in silico digest), and *f*(*q*) = peptide length correction factor.

The peptide-length correction factor takes into account the technical bias of the MS instrument, which resulted in certain peptide lengths being observed more often than others. This was learned from the data by comparing the observed peptide-length spectrum with the expected, and was corrected accordingly (similar to [22]). In our hands, peptide length proved to be the most important determinant of peptide observability, since using the original APEX implementation (“absolution protein expression profiling”) [22] or a retrained version of the same classifier, did not further improve the observed cross-organism abundance correlation between C. elegans and D. melanogaster (*R_S_* = 0.78).

A relative protein abundance of 1 means that the total number of amino acids in the identified peptides equals the number of amino acids in the protein. Whenever a peptide could be assigned to several proteins (because of identical predicted tryptic peptides), the amino acids were assigned fractionally. Peptides specific for any of the splice isoforms originating from a given locus were pooled. This approach means that the unit of interest in our comparisons is the gene locus—not individual splice isoforms—consistent with the observed lack of conservation of alternative splicing at very large evolutionary distances [61]. Finally, protein abundances were normalized to total amount of protein detected. To plot the data, orthologs were binned into eight groups of equal size (sorting for binning was *x* + *y*), and the means, as well as first and third quartiles, for each group were calculated. For the comparison of gene and protein expression, SAGE data for C. elegans [27] were downloaded from http://tock.bcgsc.bc.ca/cgi-bin/sage160. In order to best reflect the developmental stages analyzed in our proteome data, we chose the stages SWN21, SWL12, SWL21, SWL32, SWL41, SWYA1, MIXED, SW022, and DAUER. Only entries with “source = coding_RNA” were considered, and the average of the nine columns was calculated. SAGE data for D. melanogaster [29] were obtained from Professor San Ming Wang (Northwestern University, Evanston, Illinois). D. melanogaster SAGE tags were mapped to all transcripts from FlyBase release 5.3. The C. elegans Affymetrix GeneChip data were obtained from the Genome British Columbia C. elegans Gene Expression Consortium at http://elegans.bcgsc.bc.ca. The D. melanogaster Affymetrix GeneChip data [28] were obtained from http://www.flyatlas.org. For 2,695 pairs of orthologs protein abundance, SAGE and Affymetrix data were compared (in case of several paralogs, only one of them had to have data from all three measurements). For the comparisons of different abundances, Spearman rank correlation coefficients were computed to avoid assumptions about the underlying distributions. Probabilities for the correlation coefficients were calculated as implemented in R; all corresponding *p-*values were better than 2.2E−16. Further supporting the validity of spectral counting as a semiquantitative measure is a comparison of C. elegans protein abundance data against protein abundance data in yeast [41]. Importantly, the latter is *not* based on MS, but on immunodetection of tagged open reading frames. Orthologs correlate linearly in their abundance over two orders of magnitude (*R_S_* = 0.54; Figure S10). The correlation for sequence conservation (aligned to D. melanogaster) and protein abundance was calculated for 4,013 C. elegans proteins. Orthologs were binned into eight groups of equal size (Figure 5C).

## Supporting Information

Figure S1 Tandem Mass Spectra of Novel Peptides The annotated MS/MS spectra of peptides from (A) T01B11.3 (SYN-4) and (B) F47B7.7.
(289 KB PDF)

Figure S2 Coordinated Expression of Operon Genes The number of detected loci per operon deviates from what would be expected under simple independence, as shown exemplary for operons of lengths 4–6 (A–C). A higher fraction of operons than expected is either fully expressed (all proteins detected) or hardly expressed at all (none or only few proteins detected).
(23 KB PDF)

Figure S3 Proteins Encoded by Operon Genes Are More Abundant Than Those of Singleton Genes, Even When Focusing Exclusively on Embryos, L1, and Dauer Larvae Although clearly significant, the effect size is lower than for the whole, presumably due to undersampling (each plot represents less than 12% of the total data). Medians are indicated as black dots, and whiskers encompass the range from 25% to 75% of values.
(775 KB PDF)

Figure S4 Comparing the Abundances of Proteins and Transcripts, Specifically in Embryos Only (Worm and Fly) (A) Protein abundances of 1,195 conserved pairs of orthologs, which were detected in embryos of both D. melanogaster and C. elegans, and for which transcript data were available (see below). Protein abundances were estimated by spectral counting (limited to data from experiments using embryos, reducing the data to about one tenth of the total).
(B) Spearman rank correlation coefficients. Protein abundances correlate better across organisms than transcript abundances, and better than protein versus transcript within organisms.
(C) Transcript abundances of the same 1,195 conserved pairs of orthologs as in (A), from published measurements using Affymetrix arrays. Raw CEL files were reanalyzed using the MBEI algorithm as implemented in the cCHIP package. C. elegans embryo data were from the Genome British Columbia C. elegans Gene Expression Consortium, and the D. melanogaster data was from the ArrayExpress database, using wild-type controls from the experiments E-GOED-2780, E-MEXP-879, and E-MEXP-623, which cover embryonic development at a number of time points ranging from 2.5 h to 19 h after egg-laying. Medians of equal-sized bins are indicated as crosses; whiskers encompass the range from 25% to 75% of values.
(430 KB PDF)

Figure S5 Down-Sampling of Proteomics Data to Ensure Independence of Peptide Counts Individually aligned pairs of orthologs were scanned for residues R and K, in order to identify aligned tryptic cleavage sites (red vertical lines). Peptide identifications were then down-sampled in alternating stretches of the alignment, to make sure that orthologous peptides are counted for one of the two organisms only. The Spearman rank correlation dropped to 0.68. Intriguingly, this result is almost identical to what is expected simply due to the reduction of the data by half (*R_S_* = 0.67 when randomly discarding 50% of the peptides); this shows that the strong correlation between C. elegans and D. melanogaster is not simply due to a tendency of orthologous peptides to be detected equally well. To also exclude local effects (i.e., dependencies between neighboring peptides), an independent test was performed for which proteins were cut in half, and N-terminal and C-terminal fragments were counted separately. In this test, when comparing orthologous proteins only via nonoverlapping halves (N-terminus versus C-terminus), the cross-organism correlation dropped to 0.66. In contrast, when comparing N-termini with N-termini (or C-termini with C-termini), the correlation was higher (0.71). This indicates that there are indeed some local dependencies between peptide counts, but not enough to explain the high interorganism correlation we observe when using the full data.
(104 KB PDF)

Figure S6 Expression Levels of Duplicated Genes Genes were classified as duplicated when an orthologous group contained more than one gene in one lineage, but only a single gene in the other lineage. Abundances of duplicated genes were either plotted separately (A), or pooled for each group (B). Columns marked with asterisks (***), are significantly different (*p-*value better than 1E−15). Medians are indicated as black dots, and whiskers encompass the range from 25% to 75% of values.
(227 KB PDF)

Figure S7 Fly Orthologs of Worm Operon Genes D. melanogaster genes were classified according to whether their orthologs in C. elegans are part of operons. Note that these genes are not organized in operons in the fly, nor are they even neighbors on the chromosome. Still, fly proteins are more abundant when their worm orthologs are arranged in operons. *p-*values: a single asterisk (*) indicates better than 1E−5; double asterisks (**) indicate better than 1E−10; and triple asterisks (***) indicate better than 1E−15. Medians are indicated as black dots, and whiskers encompass the range from 25% to 75% of values.
(174 KB PDF)

Figure S8 Statistical Bias Analysis of the Protein Parameters Length, pI, and Hydrophobicity Distributions of the parameters of the identified proteins versus all proteins in WormBase (WS140). Overrepresented areas are shown in green, underrepresented areas in yellow (*p-*values were better than 1E−10; for details about the applied statistics, see [10]).
(19 KB PDF)

Figure S9 The Predicted C. elegans Transmembrane Proteome and Its Molecular Function We predicted the transmembrane topology of the entire C. elegans proteome and included the molecular function of the proteins with transmembrane helices. The percentages are referring to the entire dataset. Proteins with a cytoplasmic C-terminus were plotted upwards; proteins with an extracytoplasmic C-terminus were plotted downwards. The color code for the molecular function is indicated.
(1.39 MB PDF)

Figure S10 Further Support for the Validity of Protein Quantification in C. elegans, from Comparison against Published S. cerevisiae Data Protein abundances deduced from spectral counting (C. elegans) and from protein tagging and immunodetection (yeast [41]) of 1,092 pairs of orthologs from both species yielded a correlation coefficient of *R_S_* = 0.54. Medians of equal-sized bins are indicated as crosses; whiskers encompass the range from 25% to 75% of values.
(143 KB PDF)

Table S1 Identified C. elegans Proteins and Peptides In our shotgun proteomic approach, 84,962 unique peptides were identified after filtering with the PeptideProphet probability score equal to or greater than 0.9. The scan numbers, the peptides, and the coding sequence of the proteins they mapped to are listed.
(8.55 MB ZIP)

Table S2 Intraspecies Protein versus Transcript Correlations, Broken Down into Functional Categories Both fly and worm proteins were mapped to GO slim categories by a similar procedure. In both organisms, comparable categories show a high or low correlation. In addition, even categories of relatively low abundance (e.g., “DNA metabolism”) can have a high correlation, indicating that the ranking is not simply based on measurement accuracy.
(39 KB PDF)

Table S3 List of Experiments The experiment ID, the developmental stages of the worm, the sample type, and the biochemical separation methods are listed.
(18 KB PDF)

## Abbreviations

FDR: false discovery rate
GO: Gene Ontology
MS: mass spectrometry
MS/MS: tandem mass spectrometry
pI: isoelectric point
SAGE: serial analysis of gene expression
