# Supplementary material for: Comparative Functional Analysis of the Caenorhabditis elegans and Drosophila melanogaster Proteomes
Source: PLoS Biol. 2009 Mar 3;7(3):e1000048. doi: 10.1371/journal.pbio.1000048 (PMC2650730; doi:10.1371/journal.pbio.1000048)
Supplement: Supplementary file 1 [file pbio.1000048.sg001.pdf]

Schrimpf et al., Figure S1

**A** SYN-4 (T01B11.3)

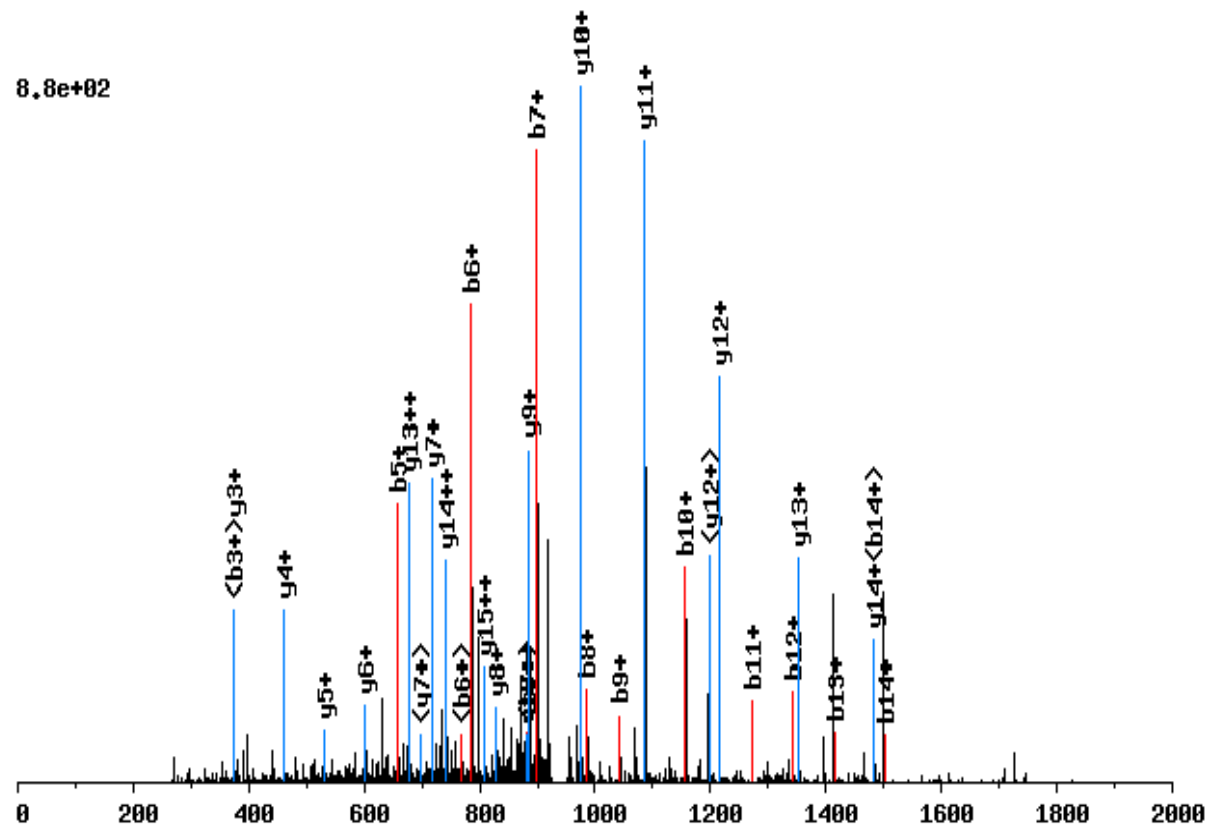


| b+ | # | AA | # | y+ | y2+ |
|---|---|---|---|---|---|
| 114.1674 | 1 | L | 17 | | |
| 261.3439 | 2 | F | 16 | 1759.9745 | 880.4912 |
| 390.4594 | 3 | E | 15 | 1612.7979 | 806.9029 |
| 521.6520 | 4 | M | 14 | 1483.6825 | 742.3452 |
| 658.7931 | 5 | H | 13 | 1352.4899 | 676.7489 |
| 786.9238 | 6 | Q | 12 | 1215.3488 | 608.1784 |
| 900.0832 | 7 | I | 11 | 1087.2181 | 544.1130 |
| 987.1614 | 8 | S | 10 | 974.0587 | 487.5333 |
| 1044.2133 | 9 | G | 9 | 886.9805 | 443.9942 |
| 1157.3728 | 10 | I | 8 | 829.9285 | 415.4682 |
| 1271.4766 | 11 | N | 7 | 716.7691 | 358.8885 |
| 1342.5554 | 12 | A | 6 | 602.6653 | 301.8366 |
| 1413.6342 | 13 | A | 5 | 531.5865 | 266.2972 |
| 1500.7124 | 14 | S | 4 | 460.5077 | 230.7578 |
| 1597.8291 | 15 | P | 3 | 373.4295 | 187.2187 |
| 1726.9446 | 16 | E | 2 | 276.3128 | 138.6604 |
| | 17 | K | 1 | 147.1973 | 74.1026 |

**B** F47B7.7

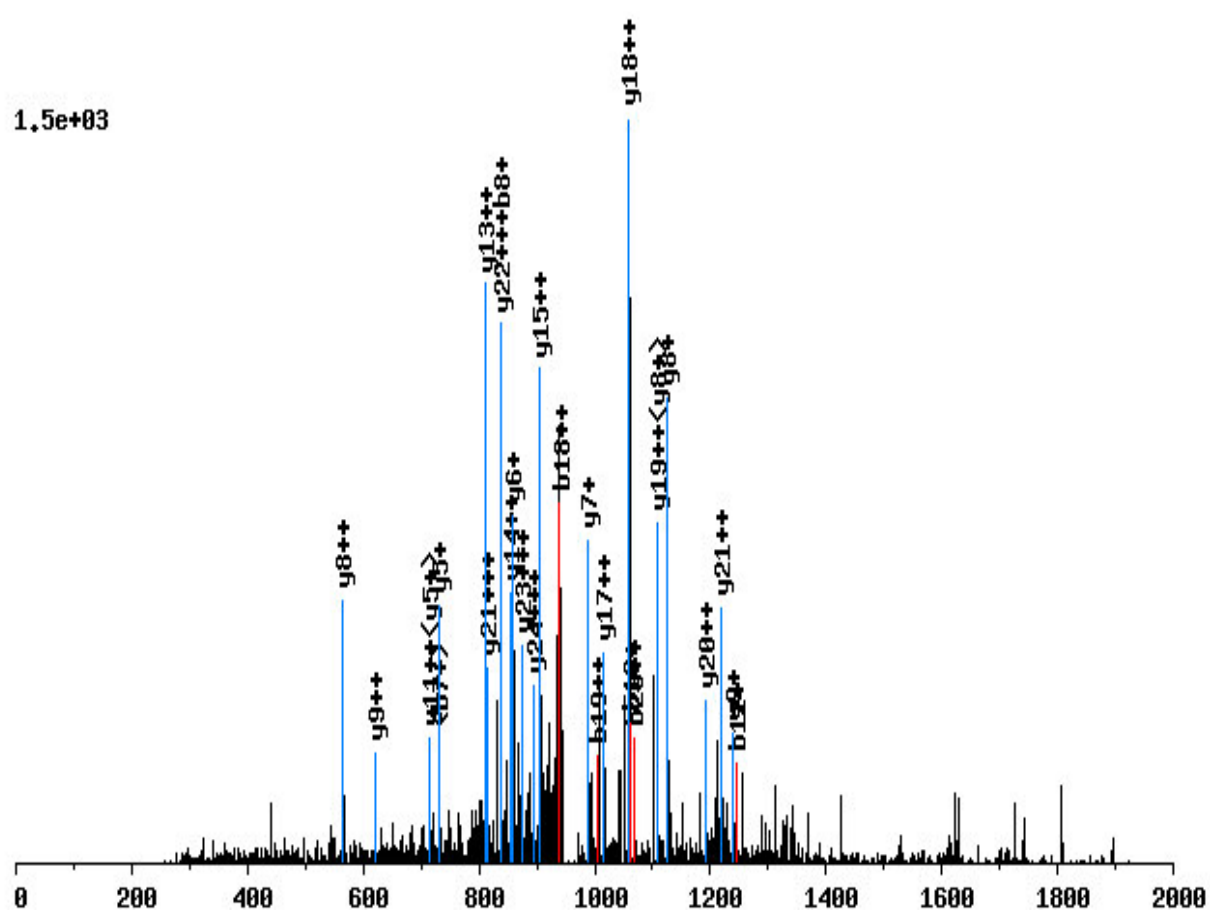


| b+ | b2+ | # | AA | # | y+ | y2+ | y3+ |
|---|---|---|---|---|---|---|---|
| 187.2211 | 94.1145 | 1 | W | 25 | | | |
| 244.2731 | 122.6405 | 2 | G | 24 | 2681.7912 | 1341.3996 | 894.6024 |
| 359.3617 | 180.1848 | 3 | D | 23 | 2624.7393 | 1312.8736 | 875.5850 |
| 430.4405 | 215.7242 | 4 | A | 22 | 2509.6507 | 1255.3293 | 837.2222 |
| 487.4924 | 244.2502 | 5 | G | 21 | 2438.5719 | 1219.7899 | 813.5292 |
| 650.6683 | 325.8381 | 6 | Y | 20 | 2381.5199 | 1191.2639 | 794.5119 |
| 749.8009 | 375.4044 | 7 | V | 19 | 2218.3440 | 1109.6760 | 740.1200 |
| 836.8791 | 418.9435 | 8 | S | 18 | 2119.2114 | 1060.1097 | 707.0758 |
| 974.0202 | 487.5141 | 9 | H | 17 | 2032.1332 | 1016.5706 | 678.0497 |
| 1061.0984 | 531.0532 | 10 | S | 16 | 1894.9921 | 948.0000 | 632.3360 |
| 1158.2151 | 579.6115 | 11 | P | 15 | 1807.9139 | 904.4609 | 603.3099 |
| 1245.2933 | 623.1506 | 12 | S | 14 | 1710.7973 | 855.9026 | 570.9377 |
| 1342.4099 | 671.7089 | 13 | P | 13 | 1623.7191 | 812.3635 | 541.9116 |
| 1443.5150 | 722.2615 | 14 | T | 12 | 1526.6024 | 763.8052 | 509.5394 |
| 1500.5669 | 750.7874 | 15 | G | 11 | 1425.4973 | 713.2526 | 475.8377 |
| 1629.6824 | 815.3452 | 16 | E | 10 | 1368.4454 | 684.7267 | 456.8204 |
| 1742.8419 | 871.9249 | 17 | I | 9 | 1239.3299 | 620.1689 | 413.7819 |
| 1879.9829 | 940.4954 | 18 | H | 8 | 1126.1705 | 563.5892 | 376.0621 |
| 2009.0984 | 1005.0532 | 19 | E | 7 | 989.0294 | 495.0187 | 330.3484 |
| 2138.2139 | 1069.6109 | 20 | E | 6 | 859.9139 | 430.4609 | 287.3099 |
| 2301.3899 | 1151.1989 | 21 | Y | 5 | 730.7984 | 365.9032 | 244.2714 |
| 2429.5206 | 1215.2643 | 22 | Q | 4 | 567.6225 | 284.3152 | 189.8794 |
| 2592.6965 | 1296.8522 | 23 | Y | 3 | 439.4917 | 220.2498 | 147.1692 |
| 2693.8016 | 1347.4048 | 24 | T | 2 | 276.3158 | 138.6619 | 92.7772 |
| | | 25 | R | 1 | 175.2107 | 88.1093 | 59.0755 |

**Figure S1**
Tandem mass spectra of novel peptides. The annotated MS/MS spectra of peptides from (A) T01B11.3 (SYN-4) and (B) F47B7.7.
