# Supplementary material for: Comparative Functional Analysis of the Caenorhabditis elegans and Drosophila melanogaster Proteomes
Source: PLoS Biol. 2009 Mar 3;7(3):e1000048. doi: 10.1371/journal.pbio.1000048 (PMC2650730; doi:10.1371/journal.pbio.1000048)
Supplement: Supplementary file 2 [file pbio.1000048.sg002.pdf]

Schrimpf et al., Figure S2

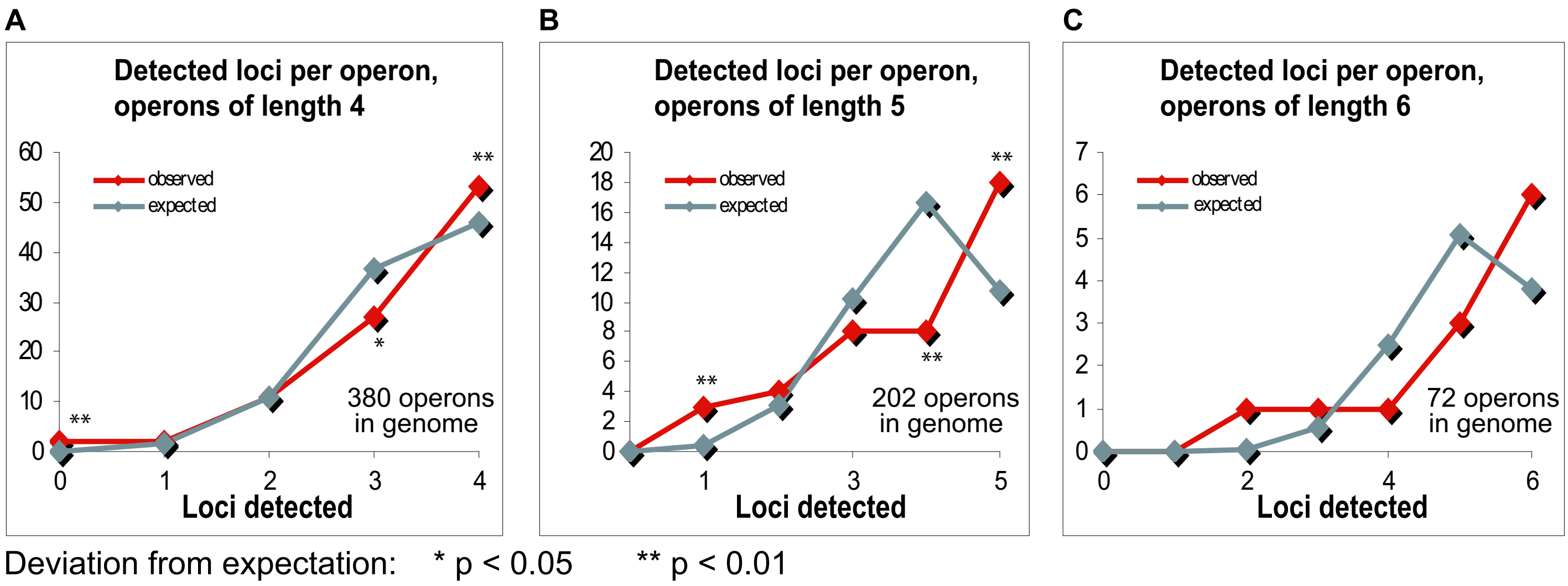

Figure S2

Coordinated expression of operon genes**.** The number of detected loci per operon deviates from what would be expected under simple independence, as shown exemplary for operons of lengths 4 - 6 (A-C). A higher fraction of operons than expected is either fully expressed (all proteins detected) or hardly expressed at all (none or only few proteins detected).
