# Supplementary material for: Comparative Functional Analysis of the Caenorhabditis elegans and Drosophila melanogaster Proteomes
Source: PLoS Biol. 2009 Mar 3;7(3):e1000048. doi: 10.1371/journal.pbio.1000048 (PMC2650730; doi:10.1371/journal.pbio.1000048)
Supplement: Supplementary file 3 [file pbio.1000048.sg003.pdf]

Schrimpf et al., Figure S3

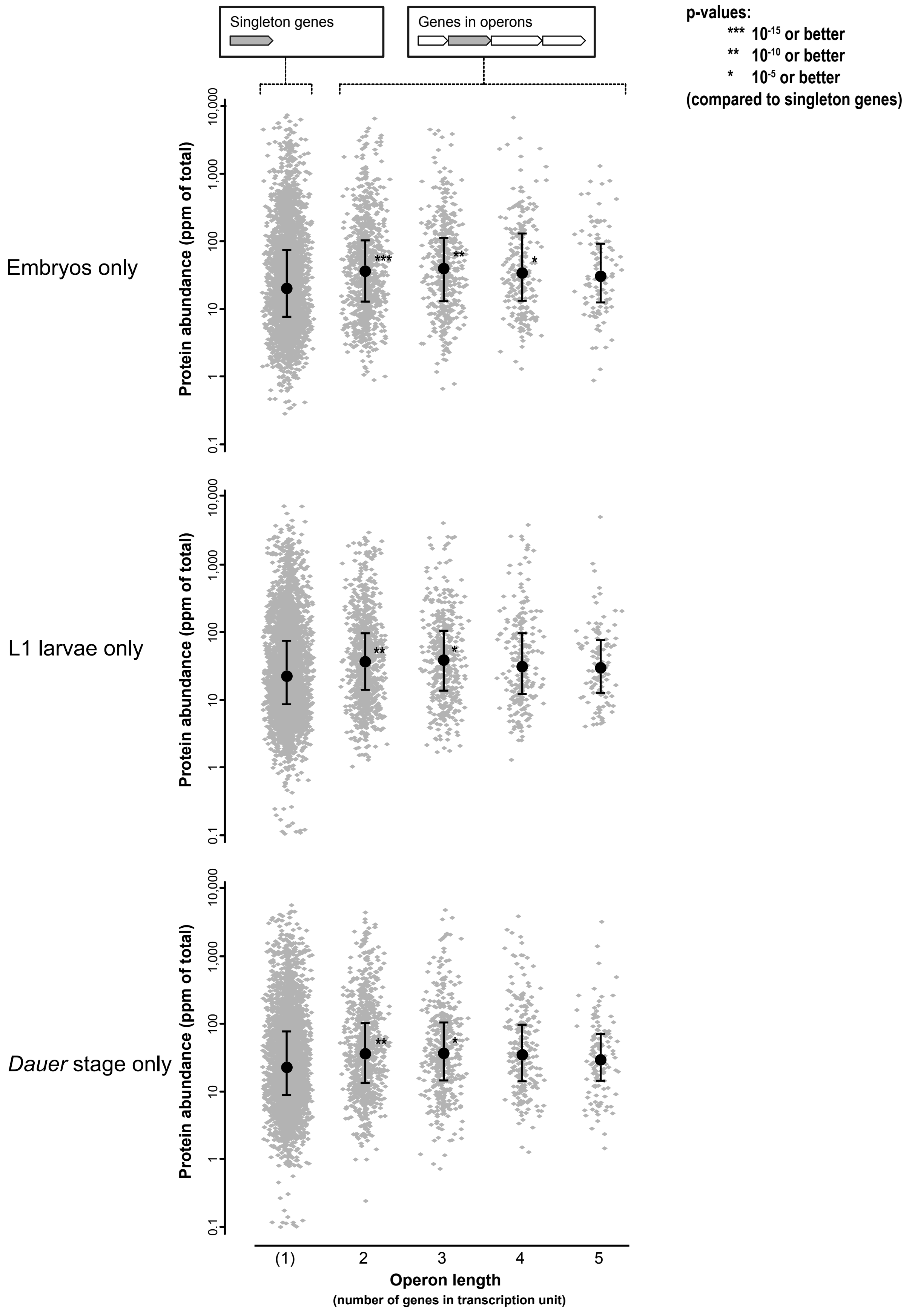

Figure S3

Proteins encoded by operon genes are more abundant than those of singleton genes, even when focusing exclusively on embryos, L1, and *Dauer* larvae. While clearly significant, the effect size is lower than for the whole, presumably due to undersampling (each plot represents less than 12% of the total data).
