# Supplementary material for: Comparative Functional Analysis of the Caenorhabditis elegans and Drosophila melanogaster Proteomes
Source: PLoS Biol. 2009 Mar 3;7(3):e1000048. doi: 10.1371/journal.pbio.1000048 (PMC2650730; doi:10.1371/journal.pbio.1000048)
Supplement: Supplementary file 4 [file pbio.1000048.sg004.pdf]

Schrimpf et.al., Figure S4

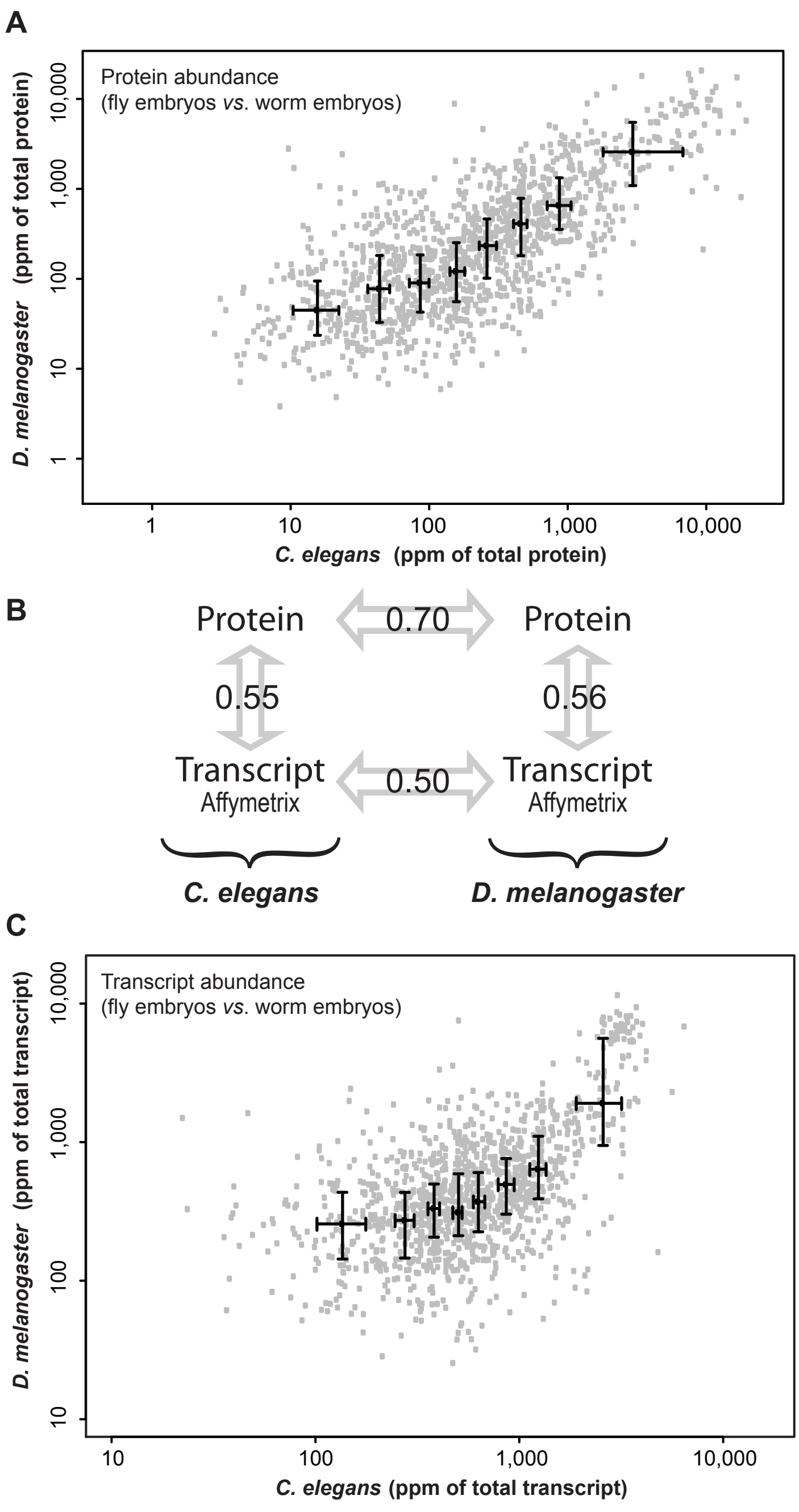

Figure S4

Comparing the abundances of proteins and transcripts, specifically in embyros only (worm and fly). (A) Protein abundances of 1,195 conserved pairs of orthologs, which were detected in embryos of both *D. melanogaster* and *C. elegans*, and for which transcript data was available (see below). Protein abundances were estimated by spectral counting (limited to data from experiments using embryos, reducing the data to about one tenth of the total). (B) Spearman's rank correlation coefficients. Protein abundances correlate better across organisms than transcript abundances, and better than protein *vs*. transcript within the organisms. (C) Transcript abundances of the same 1,195 conserved pairs of orthologs as in (A), from published measurements using Affymetrix arrays. Raw CEL-files were re-analyzed using the MBEI algorithm as implemented in the dCHIP package. *C. elegans* embryo data were from the Genome British Columbia *C. elegans* Gene Expression Consortium, and *D. melanogaster* data were from the ArrayExpress database, using wildtype controls from the experiments E-GOED-2780, E-MEXP-879, and E-MEXP-623, which cover embryonic development at a number of time points ranging from 2.5 h to 19 h after egg-laying.
