# Supplementary material for: Comparative Functional Analysis of the Caenorhabditis elegans and Drosophila melanogaster Proteomes
Source: PLoS Biol. 2009 Mar 3;7(3):e1000048. doi: 10.1371/journal.pbio.1000048 (PMC2650730; doi:10.1371/journal.pbio.1000048)
Supplement: Supplementary file 5 [file pbio.1000048.sg005.pdf]

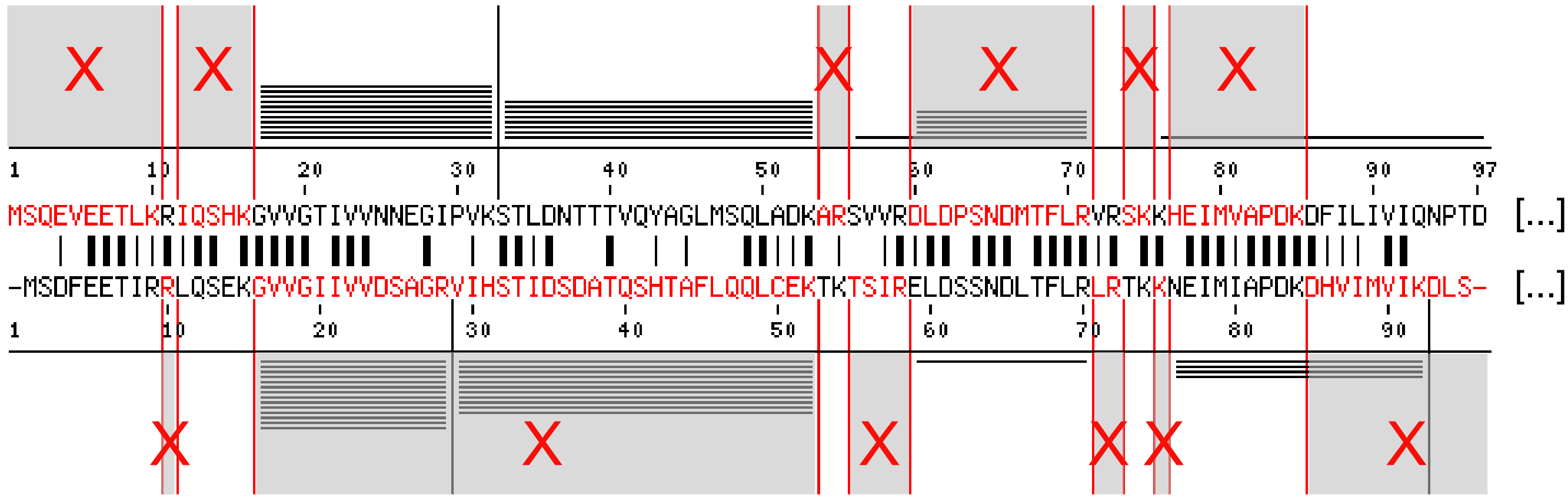

Figure S5
Down-sampling of proteomics data to ensure independence of peptide counts. Individually aligned pairs of orthologs were scanned for residues R and K, in order to identify aligned tryptic cleavage sites (red vertical lines). Peptide identifications were then down-sampled in alternating stretches of the alignment, to make sure that orthologous peptides are counted for one of the two organisms only. The Spearman's rank correlation dropped to 0.68. Intriguingly, this result is almost identical to what is expected simply due to the reduction of the data by half (R_S=0.67 when randomly discarding 50% of the peptides); this shows that the strong correlation between *C. elegans* and *D. melanogaster* is not simply due to a tendency of orthologous peptides to be detected equally well. To also exclude local effects (i.e. dependencies between neighboring peptides), an independent test was performed for which proteins were cut in half, and N-terminal and C-terminal fragments were counted separately. In this test, when comparing orthologous proteins only via non-overlapping halves (N-terminus *vs*. C-terminus), the cross-organism correlation dropped to 0.66. In contrast, when comparing N-termini with N-termini (or C-termini with C-termini), the correlation was higher (0.71). This indicates that there are indeed some local dependencies between peptide counts, but not enough to explain the high inter-organism correlation we observe when using the full data.
