# Supplementary material for: Comparative Functional Analysis of the Caenorhabditis elegans and Drosophila melanogaster Proteomes
Source: PLoS Biol. 2009 Mar 3;7(3):e1000048. doi: 10.1371/journal.pbio.1000048 (PMC2650730; doi:10.1371/journal.pbio.1000048)
Supplement: Supplementary file 6 [file pbio.1000048.sg006.pdf]

Schrimpf et al., Figure S6

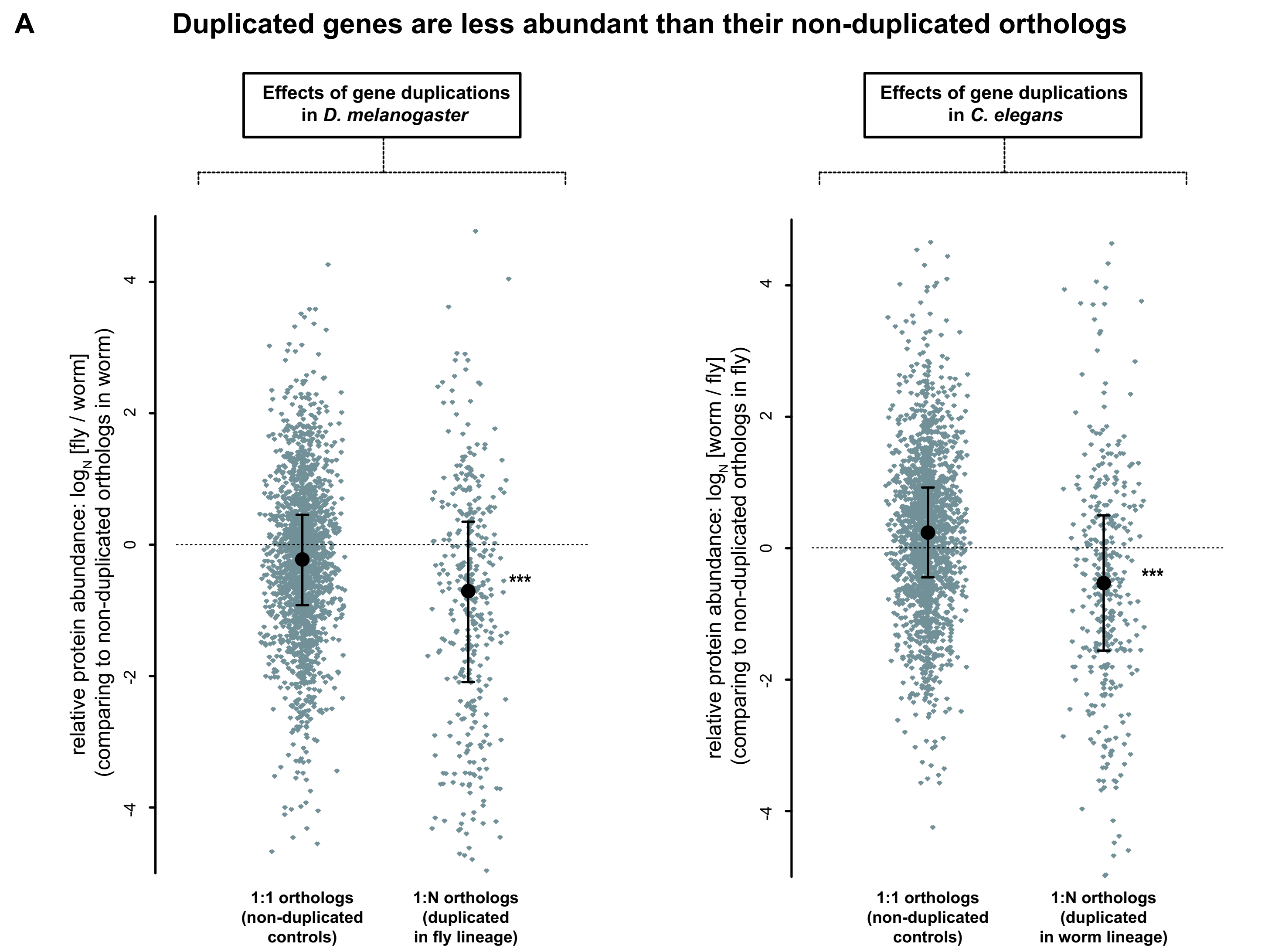

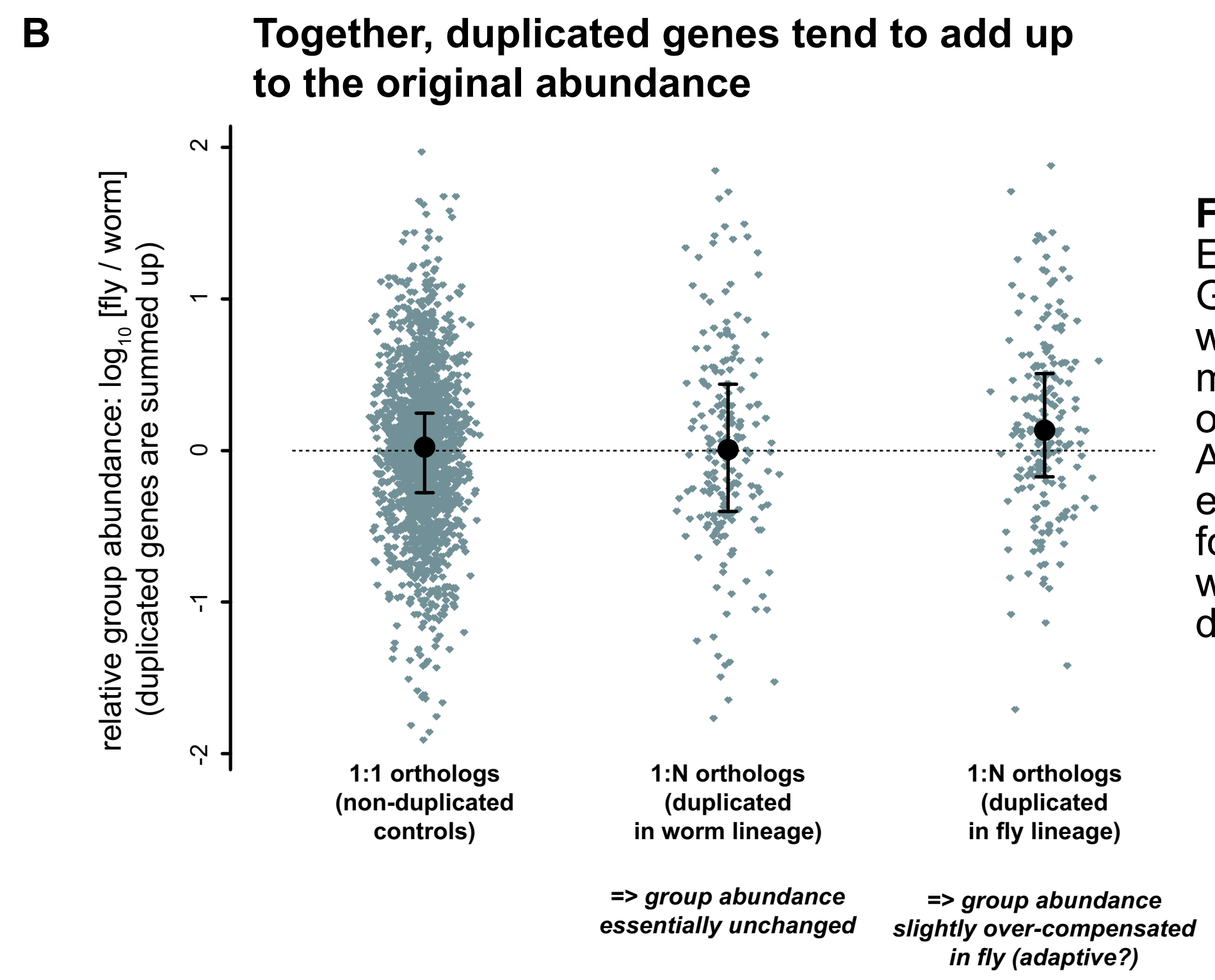

Figure S6
Expression levels of duplicated genes. Genes were classified as duplicated when an orthologous group contained more than one gene in one lineage, but only a single gene in the other lineage. Abundances of duplicated genes were either plotted separately (A), or pooled for each group (B). Columns marked with asterisks (***), are significantly different (p-value better than 1e-15).
