# Supplementary material for: Comparative Functional Analysis of the Caenorhabditis elegans and Drosophila melanogaster Proteomes
Source: PLoS Biol. 2009 Mar 3;7(3):e1000048. doi: 10.1371/journal.pbio.1000048 (PMC2650730; doi:10.1371/journal.pbio.1000048)
Supplement: Supplementary file 7 [file pbio.1000048.sg007.pdf]

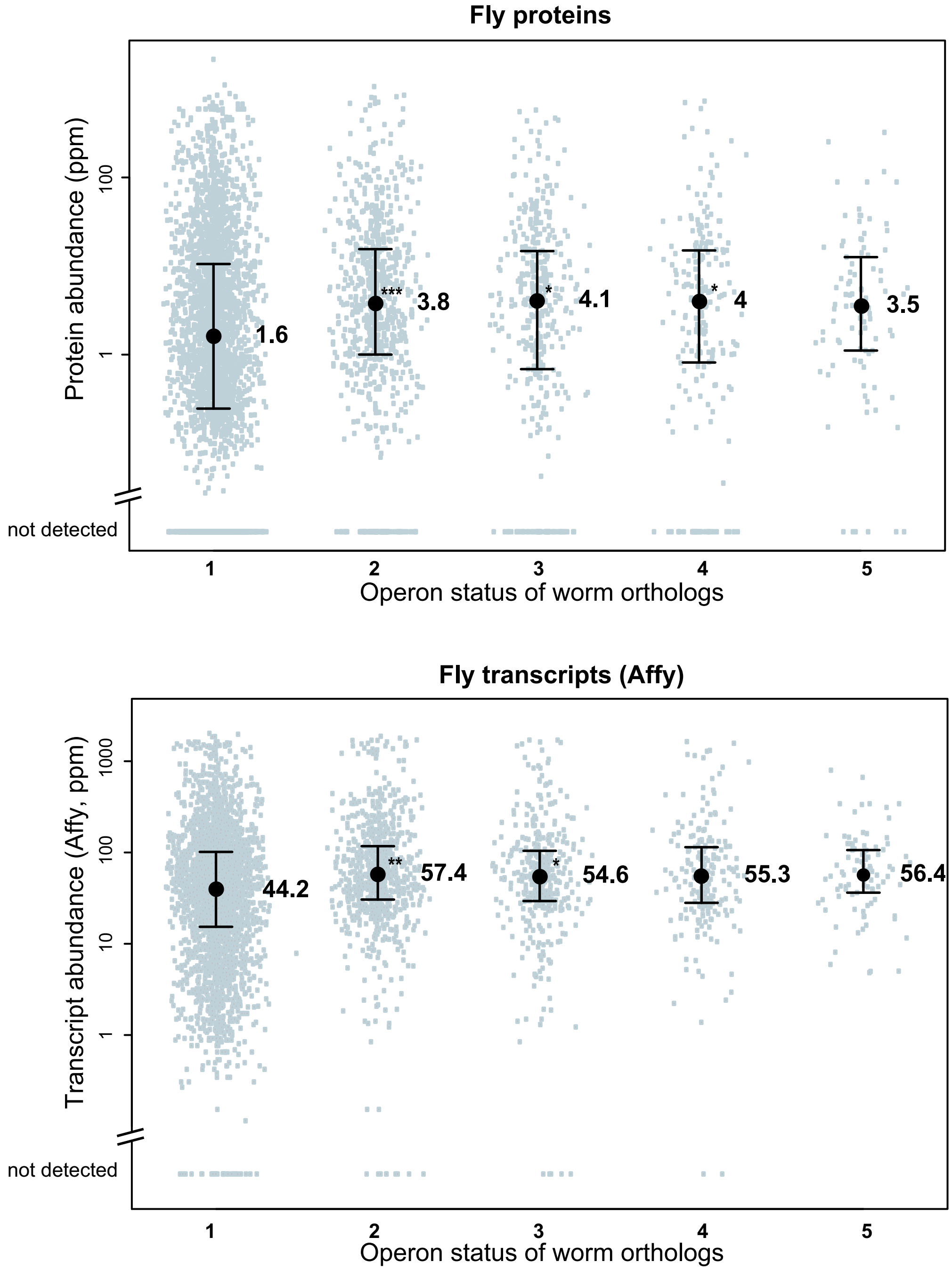

Figure S7
Fly orthologs of worm operon genes
*D. melanogaste*r genes were classified according to whether their orthologs in *C. elegans* are part of operons. Note that these genes are not organized in operons in the fly, nor are they even neighbors on the chromosome. Still, fly proteins are more abundant when their worm orthologs are arranged in operons. p-values: (**) better than 1e-10; (***) better than 1e-15.
