# Supplementary material for: Comparative Functional Analysis of the Caenorhabditis elegans and Drosophila melanogaster Proteomes
Source: PLoS Biol. 2009 Mar 3;7(3):e1000048. doi: 10.1371/journal.pbio.1000048 (PMC2650730; doi:10.1371/journal.pbio.1000048)
Supplement: Supplementary file 8 [file pbio.1000048.sg008.pdf]

Schrimpf et al., Figure S8

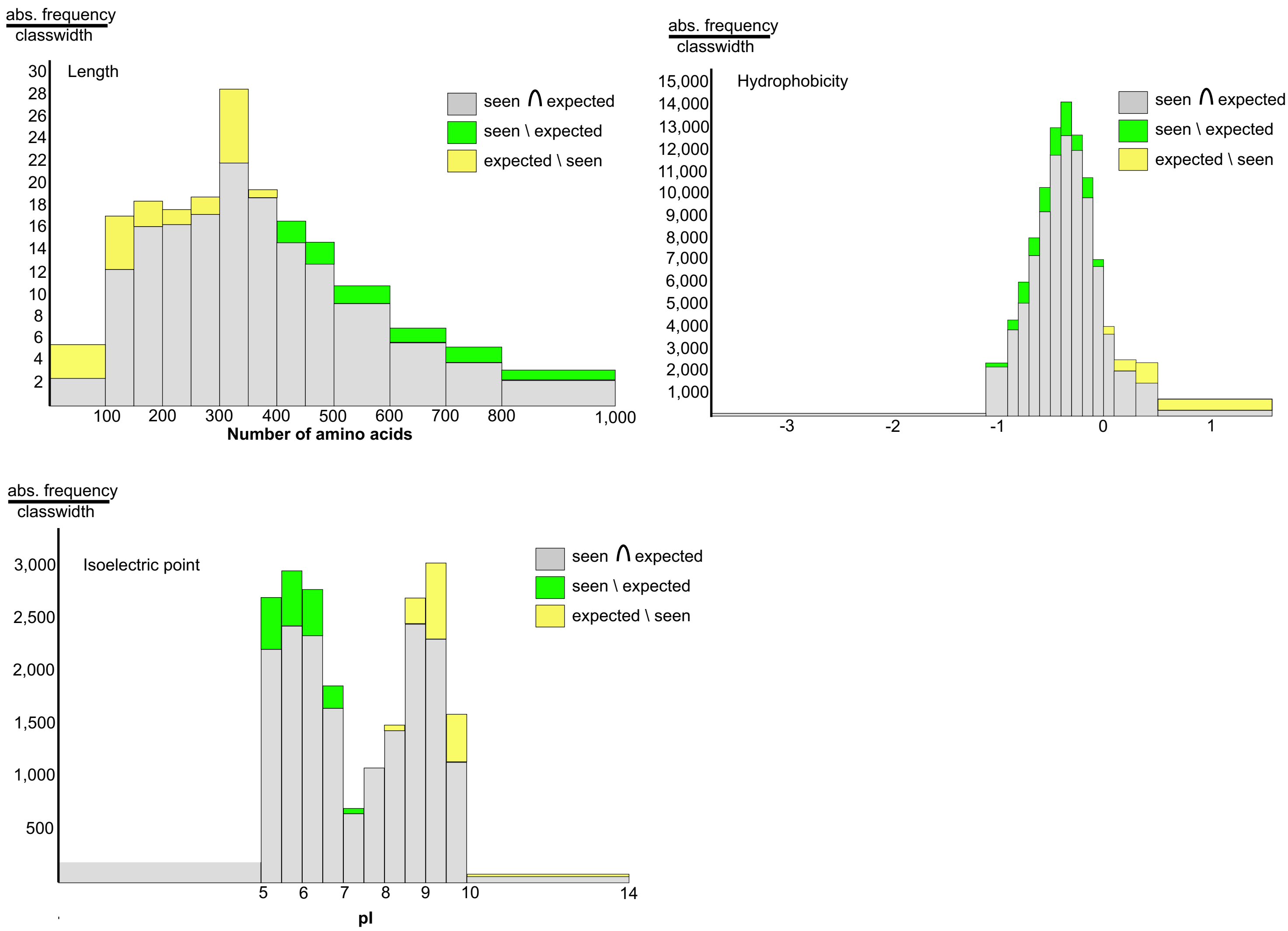

Figure S8
Statistical bias analysis of the protein parameters length, pI, and hydrophobicity. Distributions of the parameters of the identified proteins *vs.* all proteins in WormBase (WS140). Overrepresented areas are shown in green, underrepresented areas in yellow (p-values were better than 1e-10, for details about the applied statistics see [10]).
