# Supplementary material for: Comparative Functional Analysis of the Caenorhabditis elegans and Drosophila melanogaster Proteomes
Source: PLoS Biol. 2009 Mar 3;7(3):e1000048. doi: 10.1371/journal.pbio.1000048 (PMC2650730; doi:10.1371/journal.pbio.1000048)
Supplement: Supplementary file 9 [file pbio.1000048.sg009.pdf]

Schrimpf et al., Figure S9

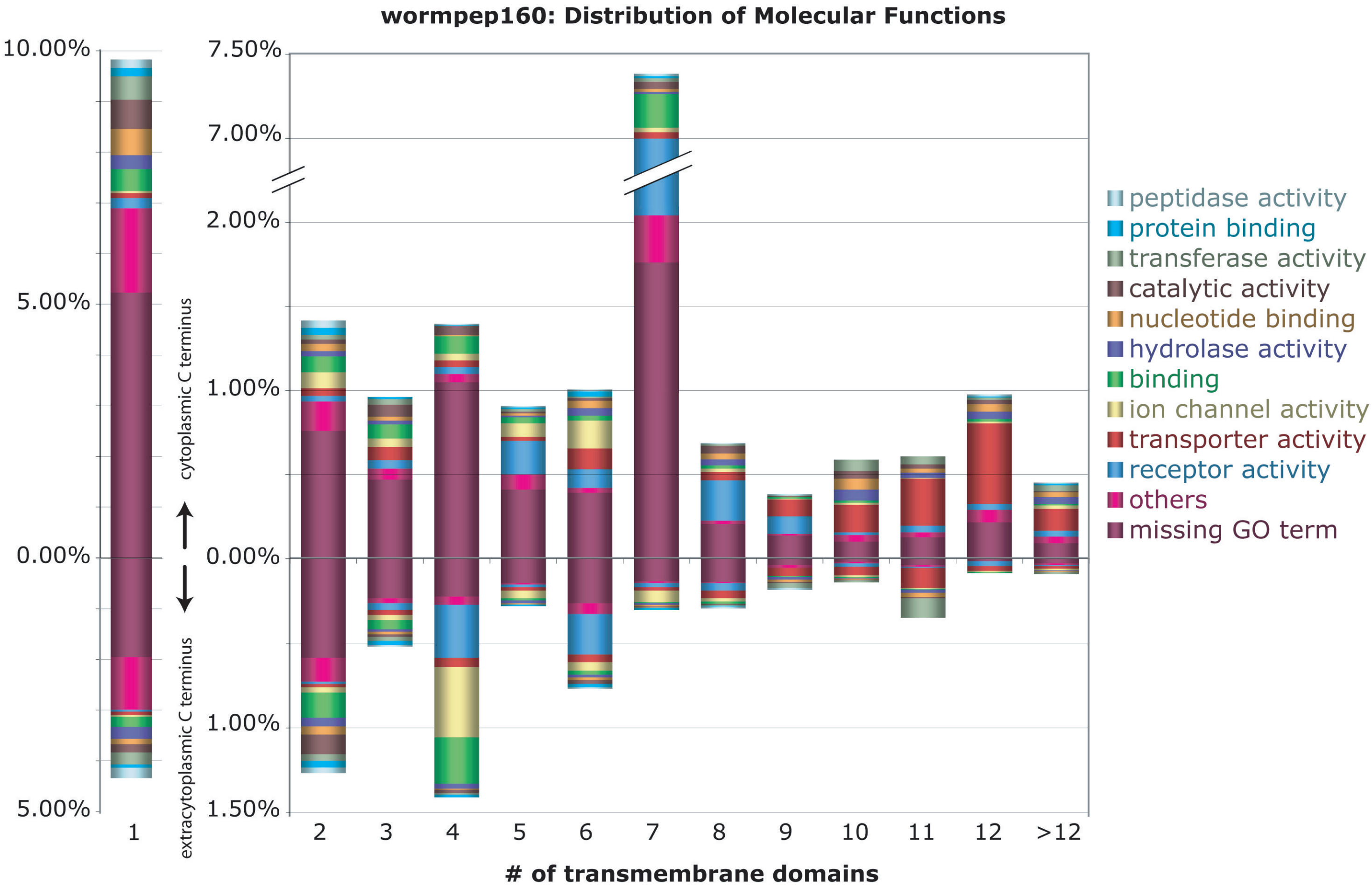

Figure S9
The predicted *C. elegans* transmembrane proteome and its molecular function. We predicted the transmembrane topology of the entire *C. elegans* proteome and included the molecular function of the proteins with transmembrane helices. The percentages are referring to the entire dataset. Proteins with a cytoplasmic C-terminus were plotted upwards, proteins with an extracytoplasmic C-terminus were plotted downwards. The colour code for the molecular function is indicated.
