# Supplementary material for: Comparative Functional Analysis of the Caenorhabditis elegans and Drosophila melanogaster Proteomes
Source: PLoS Biol. 2009 Mar 3;7(3):e1000048. doi: 10.1371/journal.pbio.1000048 (PMC2650730; doi:10.1371/journal.pbio.1000048)
Supplement: Supplementary file 10 [file pbio.1000048.sg010.pdf]

Schrimpf et al., Figure S10

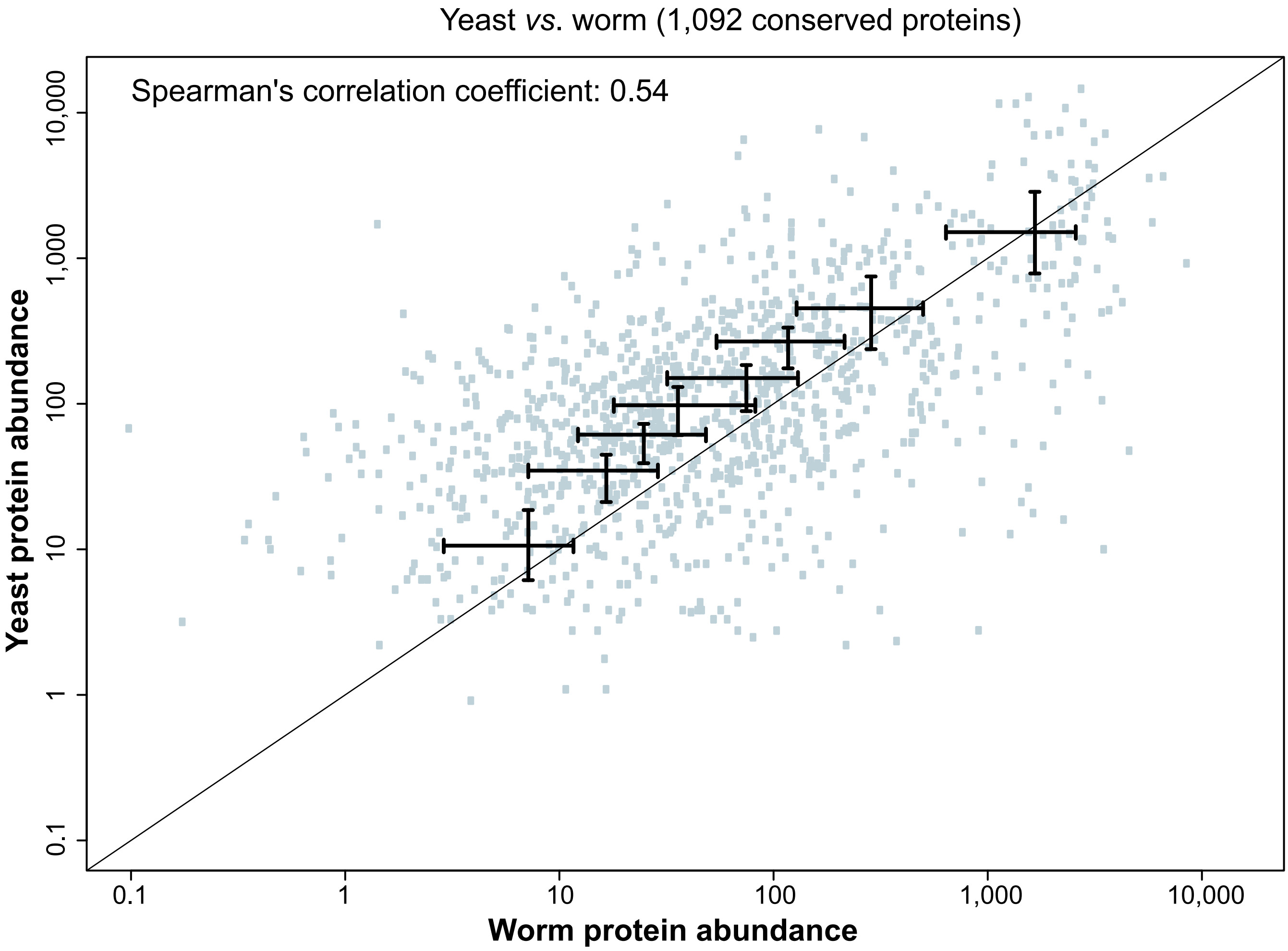

Figure S10
Further support for the validity of protein quantification in *C. elegans,* from comparison against published *Saccharomyces cerevisiae* data**.** Protein abundances deduced from spectral counting (*C. elegans*) and from protein tagging and immunodetection (yeast [41]) of 1,092 pairs of orthologs from both species yielded a correlation coefficient of R_S=0.54. Medians of equal sized bins are indicated as crosses, whiskers encompass the range from 25 to 75% of values.
