# Supplementary material for: Comparative Functional Analysis of the Caenorhabditis elegans and Drosophila melanogaster Proteomes
Source: PLoS Biol. 2009 Mar 3;7(3):e1000048. doi: 10.1371/journal.pbio.1000048 (PMC2650730; doi:10.1371/journal.pbio.1000048)
Supplement: Supplementary file 11 [file pbio.1000048.st002.pdf]

D. melanogaster, based on 8,393 detected out of 14,440 genes

all p-values below 2e-16

	***D. melanogaster* protein *vs.* affymetrix**	**Median abundance (ppm)**	
Spearman	**GO (# of entries)**	**Protein**	**Affy**
0.85	cellular macromolecule metabolic process (18)	303.7	153.3
0.82	translation (260)	174.7	142.3
0.76	generation of precursor metabolites and energy (43)	470.5	345.3
0.75	cytoskeleton organization and biogenesis (465)	43.3	67.7
0.73	cell cycle (409)	26.3	62.7
0.72	cell homeostasis (36)	229.9	93.7
0.72	ion transport (93)	12.5	36
0.69	DNA metabolism (134)	16.5	50.4
0.69	cellular component organization and biogenesis (693)	19.2	53.7
0.69	nucleobase, nucleoside, nucleotide and nucleic acid metabolism (274)	43.8	53.9
0.68	amino acid and derivative metabolism (101)	81.2	76.2
0.68	mitochondrion organization and biogenesis (54)	30.3	75.3
0.67	cellular protein metabolism (284)	19.2	90.6
0.66	transport (702)	23	53.5
0.65	metabolism (992)	25.5	61.7
0.64	developmental process (262)	13.6	43.6
0.64	multicellular organismal development (1022)	12.9	49
0.63	multicellular organismal process (447)	13.7	40.9
0.62	cell differentiation (661)	12.9	49.6
0.62	regulation of gene expression, epigenetic (69)	16.9	53.6
0.62	response to abiotic stimulus (136)	15.8	47.5
0.61	cellular process (866)	19.9	54.5
0.6	lipid metabolism (110)	13.1	61.7
0.6	response to stress (239)	10	46.9
0.59	cellular metabolism (171)	14.6	57.8
0.59	organelle organization and biogenesis (318)	31.9	59.8
0.59	protein modification (378)	11.7	49.7
0.58	behavior (227)	12.4	51.9
0.58	cell death (140)	14.5	64.3
0.58	embryonic development (300)	8.9	47.3
0.58	protein transport (117)	53.4	67.3
0.57	biological_process (343)	7	26.8
0.57	cell proliferation (125)	14.7	64.2
0.57	cellular biosynthetic process (460)	16.5	46.3
0.56	reproduction (468)	13.5	57.7
0.56	signal transduction (431)	7.1	40.6
0.56	transcription (372)	12.2	41.9
0.53	homeostatic process (63)	10.9	49.6
0.53	response to stimulus (86)	12.5	42.1
0.52	missing GO term (4556)	8.2	34.8
0.49	protein localization (92)	26.7	54.7

***C. elegans*, based on 8,501 detected out of 19,336 genes**

all p-values below 2e-16

	***C. elegans* protein *vs*. affymetrix**	**Median abundance (ppm)**	
Spearman	**GO (# of entries)**	**Protein**	**Affy**
0.91	ribosome biogenesis (12)	34.4	68.5
0.86	translation (187)	179.1	198.5
0.8	nucleobase, nucleoside, nucleotide and nucleic acid metabolism (247)	44.4	85
0.78	catabolism (134)	60.6	102.7
0.76	carbohydrate metabolism (134)	28.2	91.3
0.76	electron transport (176)	29.3	60.6
0.73	cytoskeleton organization and biogenesis (57)	25.5	54.6
0.73	protein metabolism (345)	30.1	70.6
0.69	metabolism (1362)	24.5	68.9
0.68	DNA metabolism (129)	10.4	63.6
0.67	biosynthetic process (254)	34	85.7
0.66	amino acid and derivative metabolism (129)	67	82.6
0.66	biological_process (88)	5	52
0.66	cellular component organization and biogenesis (111)	51.4	88.1
0.66	organelle organization and biogenesis (79)	35.1	75.6
0.63	ion transport (296)	2.8	41.9
0.63	transport (860)	8.7	50.8
0.62	protein transport (97)	57.5	88.3
0.57	protein modification (359)	6.2	49.2
0.54	missing GO term (5251)	8.3	49.4
0.53	transcription (322)	4.2	47.6
0.52	signal transduction (296)	3.6	34.2
0.37	lipid metabolism (129)	8.6	60

Table S2
Intra-species protein *vs.* transcript correlations, broken down into functional categories.
Both fly and worm proteins were mapped to GO slim categories by a similar procedure. In both organisms, comparable categories show a high or low correlation. In addition, even categories of relatively low abundance (e.g. 'DNA metabolism') can have a high correlation, indicating that the ranking is not simply based on measurement accuracy.
