# Supplementary material for: Comparative Functional Analysis of the Caenorhabditis elegans and Drosophila melanogaster Proteomes
Source: PLoS Biol. 2009 Mar 3;7(3):e1000048. doi: 10.1371/journal.pbio.1000048 (PMC2650730; doi:10.1371/journal.pbio.1000048)
Supplement: Supplementary file 12 [file pbio.1000048.st003.pdf]

Schrimpf et al., Table S3

Experiment ID	Worm developmental stage	Sample type	Separation method
1	mixed	membrane and soluble proteins	cICAT (cleavable isotope coded affinity tag)
2	L1	cytoplasm	cICAT, one tag
4	Dauer	cytoplasm	cICAT, one tag
5	Dauer	membrane	cICAT, one tag
7	L1	membrane	cICAT, one tag
8; 11	mixed	homogenate	protein FFE (free flow electrophoresis), selected fractions
9	mixed	cytoplasm	protein FFE, selected fractions
10	adults (Dicer knock down)	homogenate	cICAT
12; 16	mixed	cytoplasm	protein FFE, selected fractions
13	mixed	homogenate	peptide FFE
14	mixed	homogenate	protein FFE, cICAT
15	adults	homogenate	peptide IEF (isoelectric focusing) - IPG strip (immobilized pH gradient)
17	embryos laid	homogenate	SCX
18	mixed	membranes	SCX
19	embryos laid	homogenate	cICAT, one tag
20	embryos bleached	homogenate	cICAT, one tag
21	mixed	homogenate	peptide IEF - IPG strip
22	young adults	homogenate	cICAT

Table S3: List of experiments. The experiment ID, the developmental stages of the worm, the sample type and the biochemical separation methods are listed.
